# The Role of miRNAs, circRNAs and Their Interactions in Development and Progression of Hepatocellular Carcinoma: An *Insilico* Approach

**DOI:** 10.3390/genes14010013

**Published:** 2022-12-21

**Authors:** Yasmeen Ishaq, Aqsa Ikram, Badr Alzahrani, Sana Khurshid

**Affiliations:** 1 Institute of Molecular Biology and Biotechnology (IMBB), University of Lahore (UOL), Lahore 54000, Pakistan; 2 Department of Clinical Laboratory Sciences, Jouf University, Sakaka 42421, Saudi Arabia; 3 Department of Molecular Biology, Virtual University of Pakistan, 1-Davis Road, Lahore 54000, Pakistan

**Keywords:** hepatocellular carcinoma, competing endogenous RNA, miRNA, circRNA, miRNA-circRNA interactions, gene expression

## Abstract

Hepatocellular carcinoma (HCC) is a type of malignant tumor. miRNAs are noncoding RNAs and their differential expression patterns are observed in HCC-induced by alcoholism, HBV and HCV infections. By acting as a competing endogenous RNA (ceRNA), circRNA regulates the miRNA function, indirectly controlling the gene expression and leading to HCC progression. In the present study, data mining was performed to screen out all miRNAs and circRNA involved in alcohol, HBV or HCV-induced HCC with statistically significant (≤0.05%) expression levels reported in various studies. Further, the interaction of miRNAs and circRNA was also investigated to explore their role in HCC due to various causative agents. Together, these study data provide a deeper understanding of the circRNA–miRNA regulatory mechanisms in HCC. These screened circRNA, miRNA and their interactions can be used as prognostic biomarkers or therapeutic targets for the treatment of HCC.

## 1. Introduction

Hepatocellular carcinoma (HCC) is one of the very common types of cancer. Globally, it is the 4th most common cause of death after lung, colorectal and gastric cancer [1]. It is responsible for 90% of primary hepatic cancer [2]. HCC is related to non-alcoholic steatohepatitis (NASH) and non-alcoholic fatty liver disease (NAFLD), and its associated mortality rate is expected to reach about 1 million in 2030 [3]. It is caused by different aetiological factors such as HBV, HCV and alcohol. miRNA’s differential expression patterns are observed with different cancers, including HCC-induced by alcoholism, HBV and HCV infections. In HCV-infected livers, miRNAs triggered different pathways involved in the cell cycle, proteasome, lipid metabolism, antigen presentation and immune response. In HBV-infected livers, miRNAs stimulated different pathways involved in DNA damage, recombination, signal transduction and apoptosis [4]. However, the physiological impact and function of miRNAs in alcoholic liver disease is still unknown [5]. In HCC development, the mTOR pathway regulated by miRNA is very significant, but Wnt, apoptosis and MAPK signaling pathways are also disrupted by the levels of miRNA expression [6].

In addition, circular RNA (circRNA) are involved in different diseases, including HCC development [7]. circRNA is a non-coding RNA molecule with a closed circular structure that is more stable than linear RNA. It can regulate the function of miRNA by acting as a competing endogenous RNA (ceRNA), binding to miRNA like a sponge to regulate the function of miRNA, thereby indirectly controlling the expression of genes [8]. They play a critical role in the development and progression of tumors via the ceRNA mechanism. It has been reported that circRNAs (functioning as ceRNAs) mediate pathogenic mechanisms in HCC development, but many unknown circRNAs and their associated mRNAs still need to be investigated [9]. In this study, we used in silico analysis to find and compare the miRNA signatures related to HCC-induced by alcoholism, HBV and HCV infection. Moreover, we investigated the circRNA–miRNA interaction in HCC development and progression. These circRNA–miRNA interactions will be used to find new diagnostic biomarkers and therapeutic targets for HCC treatment in the future.

## 2. Materials and Methods

**Figure 1:**
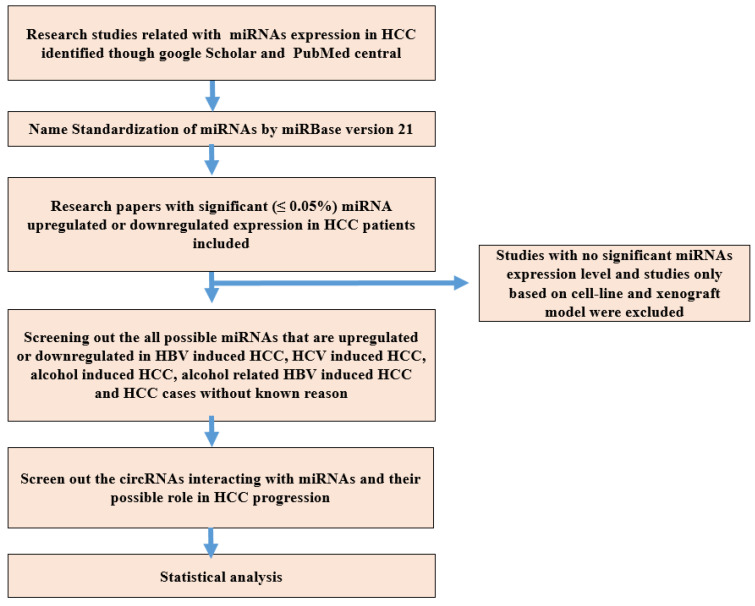
Schematic presentation of the adopted methodology.

The present study was categorized into five parts, each with its own goal: (1) To screen out upregulated and downregulated miRNAs in HBV-induced HCC reported studied through data mining; (2) to screen out upregulated and downregulated miRNAs in HCV-induced HCC reported studied through literature mining; (3) to isolate the upregulated and downregulated miRNAs in alcohol-induced HCC reported studied through data retrieval; (4) to isolate all possible upregulated and downregulated miRNAs causing HCC reported that have been studied through literature mining; and (5) to screen out the circRNAs interacting with miRNAs and their possible role in HCC progression as reported and studied through data retrieval. Strict criteria were used to screen out only those miRNA showing statistically significant (less than 0.05 was considered significant) expression in HCC reported in the included studies. The methodology followed in this study is presented in Figure 1.

### 2.1. Research Strategy

Research papers based on miRNAs’ involvement in HCC development published from 2007 to 2022 were searched using PubMed Central (PMC, https://www.ncbi.nlm.nih.gov 15 March–25 September 2022, and Google Scholar (https://scholar.google.com). We have used different terminologies to search data such as (microRNA*, miRNA*) AND ((liver AND (cancer* tumor* OR carcinoma*)) OR ((hepato* AND (cancer* tumor* OR carcinoma*)) AND ((HBV* AND (liver cancer* OR hepatic cancer* OR hepatocellular carcinoma* OR HCC*)) AND ((HCV* AND (hepatocellular OR carcinoma* OR HCC* liver cancer* OR hepatic cancer*)) AND ((Alcohol* AND (hepatocellular OR carcinoma* OR HCC* liver cancer* OR hepatic cancer*)) AND ((Alcohol* AND hepatitis AND (hepatocellular OR carcinoma* OR HCC* liver cancer* OR hepatic cancer*)).

### 2.2. Name Standardization of miRNA

To perform a miRNA expression comprehensive integrated analysis, it is important to follow the same nomenclature so that the names of miRNA can be comparable across the different studies. According to miRBase version 21 (http://www.mirbase.org/ 15 March–25 September 2022), the names of all miRNA were standardized [10]. Various conventional “major” miRNA names have been renamed in the main text according to miRBase database version 21. Non-miRNA probes and viral miRNAs were not included in the analysis.

### 2.3. Inclusion and Exclusion Criteria of Research Studies

The screened miRNAs with statistically significant (≤0.05%) expression levels between the healthy control samples and the HCC patients’ samples were retrieved from various research studies. This study included only original research papers, and the full text of each research was carefully considered. Publications related to the expression of miRNA between healthy individuals and HCC patients were further investigated. The main focus for this study is on the research articles highlighting the role of miRNA deregulation in HCC development. The articles related to all of the key players involved in miRNA deregulation in HCC development (such as HBV, HCV and alcohol) have been identified and were included in this study. Studies based on miRNA expressions in humans were included, while studies based on miRNA expressions in cell lines were not included in the present study.

### 2.4. CircRNAs and Their Target miRNAs Interaction in HCC

We screened out the circRNAs from different studies that targeted miRNAs and found out their possible interaction in HCC-related studies. We discussed the influence of circRNAs on miRNAs as well as the expression of different proteins and downstream pathways involved in HCC development and progression.

### 2.5. Data Collection

We have strictly followed those studies where the significant expression level of miRNAs was < 0.05. The lists of miRNAs with aberrant expression levels were made on the basis of included research analysis.

## 3. Results

### 3.1. Characteristics of Search Data

According to the inclusion criteria, 76 autonomous full-text research papers were retrieved from PubMed Central (PMC, https://www.ncbi.nlm.nih.gov 15 March–25 September 2022) and Google Scholar (https://scholar.google.com 15 March–25 September). These research papers reported the expression of different 88 miRNAs in HCC. This study includes all of the miRNA reported in HBV-induced HCC, HCV-induced HCC and alcohol-induced HCC. In these studies, the miRNA expression level was either checked by using RT-PCR or microarray (in some studies, both methods were used). Based on these criteria, we screened out 88 miRNA involved in the development of HCC reported in 76 studies. In addition, only those studies that reported on the significant expression of miRNA in HCC were used. Among them, 18 studies were based on HBV-induced HCC, 9 studies were on HCV-induced HCC, 4 studies focused on alcohol-induced HCC and 45 studies did not represent the causative agent of HCC. All the included studies with reported miRNA are mentioned in Table 1. miRNAs related with HBV, HCV and alcohol induced HCC (Supplementary Table S1).

Exosomes are tiny lipid-bilayer membrane structures with a diameter of 30–100 nm [87]. They can fuse with particular receptor cells and act as the main player in the cells and their micro-environments in inter-cellular communication by horizontal information transfer [88]. As special carriers of signals and materials, exosomes contain cell-specific proteins and peptides, DNAs, mRNAs, miRNAs, other non-coding RNAs and lipids, and exosomes participate in cell communication, migration, angiogenesis, tumor development and other physiological and pathological processes [89]. It has been reported by different studies that cancer cells release high levels of exosomes and contribute to tumor development [90].

The same process involves producing miRNAs and exosomal miRNAs, but miRNAs packed in exosomes are called exosomal miRNAs [91]. Current studies revealed that miRNAs mediated via exosomes also play a critical role in the development of liver cancer. These exosomal miRNAs can act as diagnostic and prognostic biomarkers of HCC [92]. HCC-related exosomal miRNAs are mentioned in the following Table 2. Exosomal miRNAs expression in HCC development (Supplementary Table S2).

**Table 2:** HCC related exosomal miRNAs.

Sr. No	miRNAs	Expression in Serum of HCC	Region	References
1	miR-10b	Upregulated	China	[93]
2	miR-18a	Upregulated	Korea	[94]
3	miR-21	Upregulated	China	[95]
4	miR-34a	Downregulated	China	[96]
5	miR-34c	Downregulated	China	[96]
6	miR-93	Upregulated	China	[97]
7	miR-122	Downregulated	USA	[98]
8	miR-195	Downregulated	Korea	[94]
9	miR-222	Upregulated	Korea	[94]
10	miR-223	Upregulated	UK	[99]
11	miR-224	Upregulated	Korea	[94]

### 3.2. Expression Profile of Identified miRNAs Involved in HCC Development

**Figure 2:**
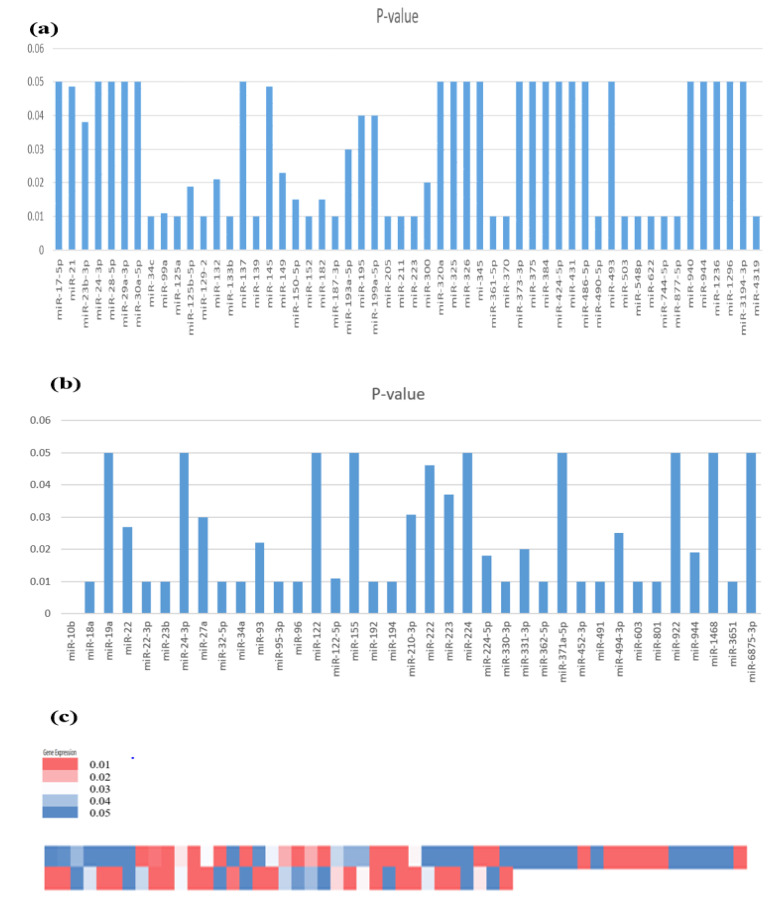
Expression profile of identified miRNAs involved in HCC. (**a**) miRNAs downregulated expression in HCC. (**b**) miRNAs upregulated expression in HCC. (**c**) Heatmap showing the miRNA’s relative downregulated and upregulated expression with *p* values < 0.05.

In the current study, 88 dysregulated miRNAs reported in 76 studies were chosen to discover their expressions in HCC. Significantly downregulated miRNAs related to HCC development are mentioned in the following figure, Figure 2A. In the Bar chart, downregulated miRNAs are listed with a significant *p*-value (<0.05) of their expression level.

Significantly upregulated miRNAs related to HCC are mentioned in Figure 2B. Overexpression of miRNAs is displayed against the *p*-value (<0.05) in the Bar chart. In addition, upregulated and downregulated miRNAs are also shown in the heatmap. High significance is represented with a darker pink color and low significance is represented with a darker blue color. The heatmap of miRNAs vs. the expression of the consistently dysregulated miRNAs in HCC patients (clustering based on significance levels). Microsoft Excel was used to build the heatmap. High significance is represented with a darker pink color and low significance is represented with a darker blue color, as shown in Figure 2C.

### 3.3. miRNA Expression in HBV-Induced HCC

Included in this study are 18 studies reporting on HBV-induced HCC showing that miR-18a, miR-19a, miR-22, miR-23b, miR-24-3p, miR-96, miR-192, miR-194, miR-210-3p, miR-222, miR-224, miR-371a-5p and miR-801 were overexpressed (Table 3, shown with a blue color). However, miR-21, miR-34c, miR-132, miR-145, miR-193a-5p, miR-205, miR-223, miR-375, miR-384, miR-548p and miR-1236 were significantly downregulated, as mentioned in Table 3, shown with a peach color.

### 3.4. miRNA Expression in HCV-Induced HCC

Included in this study are nine studies reporting HCV-induced HCC, which reveal that miR-27a, miR-122-5p, miR-155, miR-224-5p, miR-331-3p and miR-494-3p were highly upregulated (Table 4, shown with a blue color), but miR-17-5p, miR-23-3p, miR-24-3p, miR-125a, miR-139, miR-152, miR-182, miR-195 and miR-223 were significantly downregulated in HCC as mentioned in Table 4, shown with a peach color.

### 3.5. miRNA Expression in Alcohol-Induced HCC

Very few studies reported alcohol-induced HCC development. Among them, four studies represent that miR-22-3p, miR-122, miR-223 and miR-944 were significantly upregulated (Table 5, shown with a blue color), but miR-125b-5p and 199a-5p were significantly downregulated in HCC tissue, as mentioned in Table 5, shown with a peach color.

### 3.6. miRNA Expression in HCC (Generalized Studies)

Most of the HCC-related studies did not represent any particular causative agent of HCC. In these studies, the reason behind the abnormal miRNAs expression related to HCC development was not mentioned. We consider these studies as general case studies of HCC. Analysis of these 45 studies exposed that miR-10b, miR-32-5p, miR-34a, miR-93, miR-95-3p, miR-330-3p, miR-362-5p, miR-452-3p, miR-491, miR-603, miR-922, miR-1468, miR-3651 and miR-6875-3p were highly upregulated (Table 6, shown with a blue color). However, miR-28-5p, miR-29a-3p, miR-30a-5p, miR-99a, miR-129-2, miR-133b, miR-137, miR-149, miR-150-5p, miR-187-3p, miR-211, miR-300, miR-320a, miR-325, miR-326, miR-345, miR-361-5p, miR-370, miR-373-3p, miR-424-3p, miR-431, miR-486-5p, miR-490-5p, miR-493, miR-503, miR-622, miR-744-5p, miR-877-5p, miR-940, miR-1296, miR-3194-3p, miR-4319 were significantly downregulated, as shown in Table 6, shown with a peach color.

### 3.7. Comparative Analysis of miRNAs Expressed in HCC

**Figure 3:**
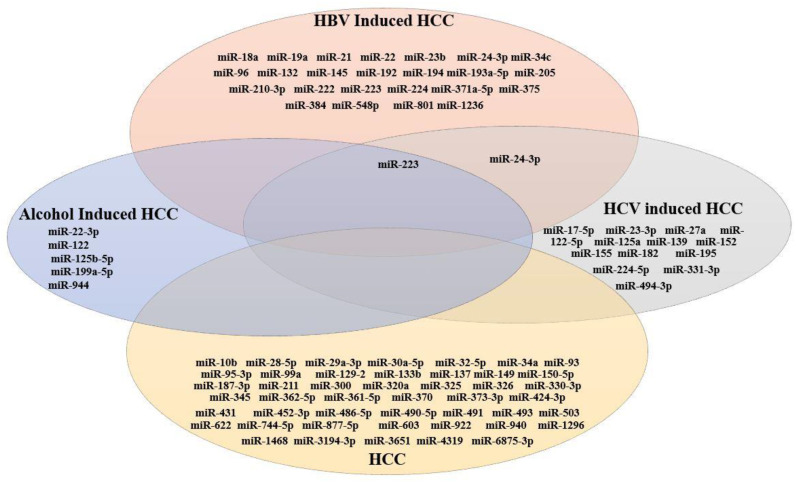
Venn diagram showing the shared miRNA profiles among HBV-induced HCC, HCV-induced HCC, alcohol-induced HCC or alcohol-related HBV and HCV-induced HCC patients and HCC patients without any known causative agent.

All possible miRNAs causing HCC are included in this study. Some miRNAs showed abnormal expression due to HBV chronic infection, HCV chronic infection and alcoholism or alcohol-related HBV infection. However, in most studies, the causative agent of abnormal miRNAs is not mentioned. In order to screen out the common miRNA causing HCC, a Venn diagram was constructed (Figure 3). Figure 3 shows all miRNAs expressed in HCC reported in various studies. In addition, the overlapped miRNA expressed in HBV, HCV and alcohol-induced HCC are mentioned in Figure 3.

Among them we screened miRNAs that were common among HBV-induced HCC, HCV-induced HCC and alcohol-induced HCC. We observed that miR-223 was commonly expressed in HCC-induced HBV, HCV and alcohol-induced HCC. However, miR-223 was downregulated in HBV and HCV-induced HCC but was significantly upregulated in alcoholism-related HCC. The dysregulation of miR-24-3p has been reported in HBV-induced HCC and HCV-induced HCC. Though, miR-24-3p was upregulated in HBV-induced HCC but downregulated in HCV-induced HCC.

### 3.8. CircRNAs and miRNAs in HBV/HCV-Induced HCC

**Figure 4:**
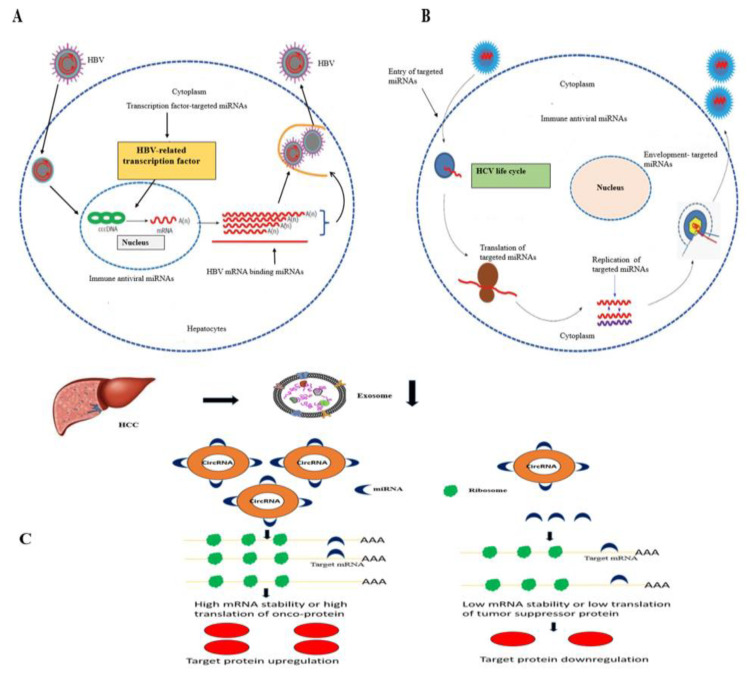
miRNAs and circRNAs involved in HBV and HCV-induced HCC. (**A**) miRNAs involved in the replication of HBV by targeting transcription factors, direct targeting viral mRNAs and regulating immune response. (**B**) miRNAs are involved in the replication of HBV via targeting viral entry, translocation, replication, envelopment and regulating of the immune response. (**C**) CircRNA crosstalk with miRNA in HCC.

It has been reported that miRNAs are involved in the gene regulation and replication of HBV/HCV by targeting different viral proteins or transcriptional factors [101]. An increasing body of evidence has revealed that HBV or HCV has developed different strategies to escape host immune surveillance and establish a persistent chronic viral infection that regulates the host’s miRNA expression, as mentioned in the Figure 4. The dysregulated miRNAs’ expression of the host via HBV or HCV infection involves in the development of different pathogenesis such as HCC (Figure 4) [102]. The mechanism of miRNA’s regulation by HBV is still not clear. Several studies report that HBx viral protein transactivates several viral and host genes, including miRNAs. HBx regulates the expression of miRNA through the transcription process via direct binding with the transcription factor, or the HBx transcript act as sponge (which particularly downregulates the miRNA via their target sequence in the viral RNA [103]. HCV infection dysregulates different miRNAs, which involves the regulation of host immune response and different cell growth signaling pathways [104].

CircRNAs are formed through back-splicing in canonical splicing machinery [105]. In eukaryotes, circRNA are mostly found in the cytoplasm and are specific to cell type. CircRNAs can interact with miRNAs via the sponging, upregulation, and downregulation of miRNAs (Figure 4). Several circRNAs participate in hepatocarcinogenesis through miRNAs sponging and disturbing the cell division, apoptosis and metastasis pathways [106].

### 3.9. CircRNA–miRNA Interaction in HCC Development

**Figure 5:**
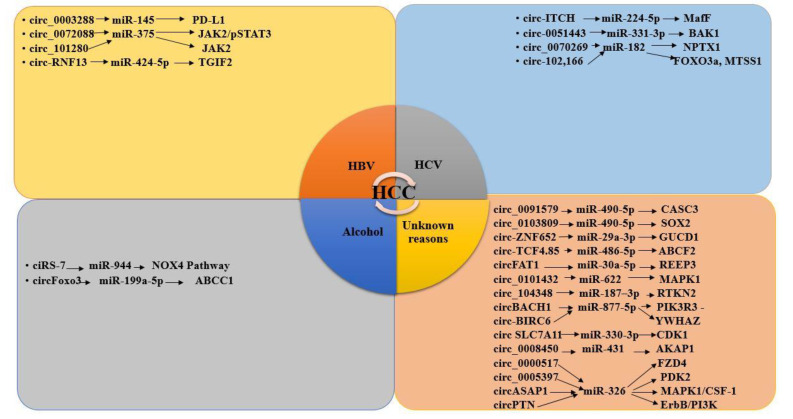
circRNA–miRNA–protein interaction in HCC.

circRNAs are non-coding RNAs; they can regulate miRNA expression by acting as ceRNA. They can upregulate, downregulate or sponge the expression of miRNAs and affect the transcription of various proteins involved in different biological pathways. Consequently, the interaction of miRNA with circRNA plays a critical role in the progression of various diseases, including HCC [8]. The circRNAs targeted the miRNAs and played a critical role in the HCC progression that has been elected in the present study. The interaction of circRNAs with their target miRNAs and proteins is shown in Figure 5.

#### 3.9.1. CircRNA–miRNA Interaction Reported in HBV-Induced HCC Studies

**Figure 6:**
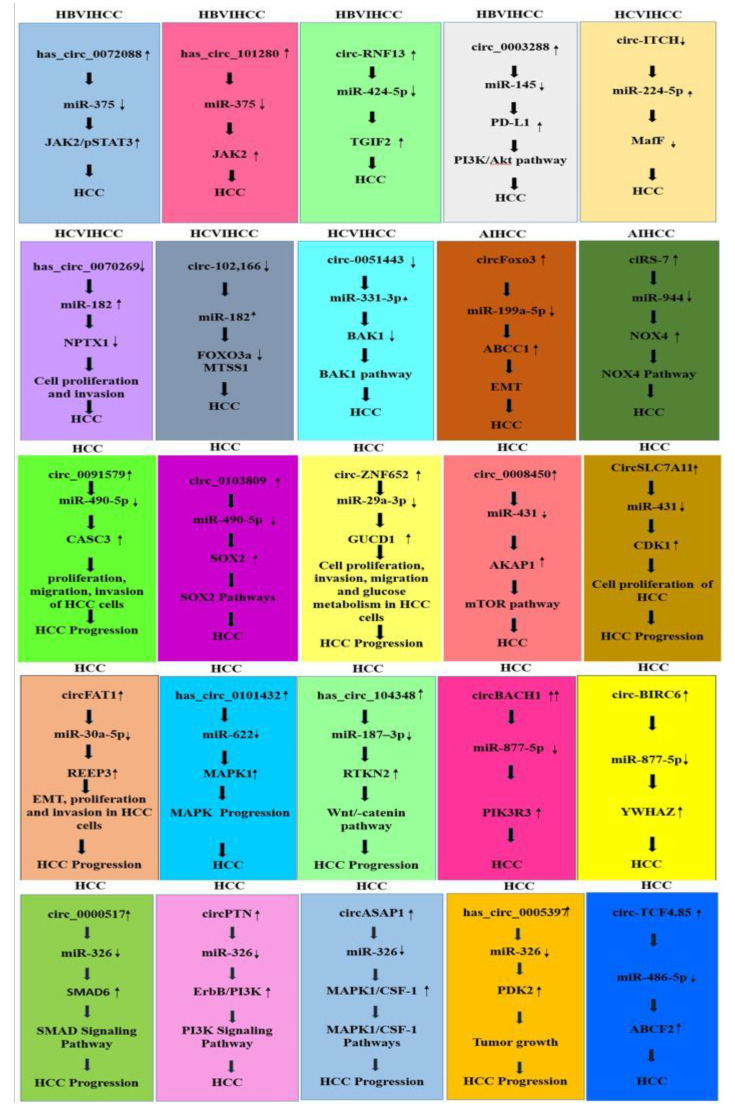
CircRNA–miRNA–mRNA interaction reported in HCC cases. HBV-induced HCC (HBVIHCC), HCV-induced HCC (HCVIHCC), Alcohol-induced HCC (AIHCC) and HCC (hepatocellular carcinoma).

HBV-induced HCC studies reported the significant interaction of circRNA–miRNA in HCC. In the present study, we have included all four studies reporting the interaction of circRNA–miRNA and their possible role in inducing HCC (Figure 6).

The overexpression of has_circ_0003288 increased the levels of programmed death-1 ligand 1 (PD-L1) protein and aided EMT migration and invasiveness in L02 cells. has_circ_0003288 served as a miR-145 sponge and directly targeted the PD-L1 3′-untranslated region (UTR) to control their expression [107]. Knockdown of has_circ_0003288 leads to the overexpression of PD-L1 and restored their EMT, migration and invasiveness. In HCC, it was discovered that the PI3K/Akt pathway mediates the metastatic phenotypes via the has_circ_0003288/PD-L1 axis [108].

Human HCC tissues with HBV infection showed overexpression of circ-RNF13, along with upregulation of TGFβ-induced factor homeobox 2 (TGIF2) and downregulation of miR-424-5p. MiR-424-5p directly interacts with circ-RNF13 [109] and TGIF2. TGIF2 is a transcriptional repressor protein [110]. Circ-RNF13 and TGIF2 acted as ceRNAs for miR-424-5p. Circ-RNF13 may act as a sponge for miR-424-5p to inhibit TGIF2 and HBV infection in HCC cells associated with HBV [109].

Has_circ_101280 promotes HCC tumorigenesis. It was reported that has_circ-101280 is overexpressed in HCC cells. Has_circ_101280 sponges the miR-375 expression, whereas miR-375 targets Janus kinase 2 (JAK2) [111]. JAK2 highly expressed in normal liver tissue of HCC may be a good prognostic biomarker for resected HCC [112]. Overall, both the in vitro and the in vivo experiments showed that has_circ_101280 significantly aided HCC tumorigenesis by sponging miR-375 and upregulating JAK2 [111].

Circ 0,072,088 is involved in cells proliferating, migrating and invading, as well as undergoing apoptosis in HCC. It was reported that circ_072088 is highly expressed in HCC tissues and cell lines. Circ_072088 targets the miR-375. miR-375 has the potential to suppress JAK2 expression further. Circ 0,072,088 functions as a molecular sponge for miR-375, activating the JAK2/STAT3 signaling pathway in a specific manner [113].

#### 3.9.2. CircRNA –miRNA Interaction in HCV-Induced HCC Studies

HCV-induced HCC reported the significant interaction of circRNA–miRNA HCC. In the present study, we have included all four studies reporting the interaction of circRNA–miRNA and their possible role in inducing HCC (Figure 6).

Circ-0051443 suppresses tumor growth in vivo. It was reported that circ-0051443 was significantly downregulated in HCC patients. Circ-0051443 competitively binds to miR-331-3p, whereas, miR-331-3p targets BCL2 Antagonist/Killer 1(BAK1) to regulate their expression, which is accompanied by an increase in BAK1 in these malignancies [114]. BAK1 is the main regulator of the cell death process and facilitates mitochondria-mediated apoptosis by protein interaction. BAK1 is linked with the development of several tumors [115]. Thus, circ_0051443 can be used as a therapeutic target and predictor for HCC [114].

Hsa_circ_0070269 acts as a tumor suppressor gene in HCC tumorigenesis. It was reported that the expression of hsa_circ_0070269 was significantly reduced in cell lines. Hsa_circ_0070269 sponges miR-182 expression and miR-182 targets the NPTX1 (neuronal pentraxin) [116]. NPTX1 belongs to the pentraxins family [117]. The upregulation of hsa_circ_0070269 inhibited HCC cell proliferation and invasion in-vitro and decreased tumor growth in vivo. NPTX1 expression was enhanced by hsa_circ_0070269 in HCC cells via sponging miR-182, which prevented aggressive tumor behavior [116].

Circ-102,166 acts as a tumor suppressor in HCC. It was reported that the expression of circ-102,166 was low in HCC patients. Circ-102,166 interacts with miR-182. However, miR-182 targets and controls the expression of a number of their downstream targets like Forkhead box O3a (FOXO3a) and Metastasis suppressor 1 (MTSS1). FOXO3a, a member of the Forkhead box O (FOXO) transcription factor family, acts as a tumor suppressor in different type of cancers [118]. MTSS1 is located in the central position of the gene function net of residual HCC [119]. In vivo and in vitro studies discovered that circ-102,166 overexpression significantly reduced the proliferation, invasion, migration and tumorigenicity of HCC cells [120].

Circ-ITCH was downregulated in HCC cells and tissues. Circ-ITCH specifically sponges miR-224-5p in HCC. miR-224-5p direct targets the MafF (MAF BZIP transcription Factor F) and reduced their expression. MafF, of the Maf family of basic leucine zipper (bZIP) transcription factors, is frequently downregulated in a variety of malignancies. The antitumor properties and expression of MafF may be controlled by the circ-ITCH/miR-224-5p axis [121].

#### 3.9.3. CircRNA–miRNA Interaction Reported in Alcohol-Induced HCC Studies

Very few studies reported on the significant interaction of circRNA–miRNA in alcohol-induced HCC. In the present study, we have included two studies reporting the interaction of circRNA–miRNA and their possible role in inducing HCC (Figure 6).

CiRS-7 stimulates the invasion, migration and progression of HCC. It was reported that ciRS-7 is highly expressed in HCC tissues. CiRS-7 acts as a microRNA sponge to control miR-944, whereas miR-944 targets and reduces the expression of NADPH oxidase 4 (NOX4) mRNA and the protein levels of the NOX4 pathway [122]. NOX4 belong to the NADPH oxidases (NOX) family and is an important component in the production of reactive oxygen species (ROS) [123]. In cells, apoptosis is induced by oxidative stress [124]. By inhibiting miR-944/NOX4, the tumor-promoting impact of ciRS-7 could be prevented [122].

It was reported that circFoxo3 expression was high in HCC tissues and cell lines. CircFoxo3 overexpression promotes HCC cell invasion and tumor growth, while circFoxo3 knockdown inhibits these processes. It was reported that circFoxo3 expression was high in HCC tissues and cell lines. CircFoxo3 interacts with miR-199a-5p and controls their expression. MiR-199a-5p directly targets the ATP binding cassette subfamily C member 1 (ABCC1) [125]. ABCC1 is also known as multidrug resistance-associated protein 1 (MRP1) and causes chemotherapy resistance in various cancers [126]. CircFoxo3 negatively regulates miR-199a-5p but positively regulates ABCC1 expression and promotes the epithelial–mesenchymal transition [125].

#### 3.9.4. CircRNA–miRNA Interaction in HCC Reported in Generalized Studies

Most of the HCC-related studies did not represent any particular causative agent of HCC and reported the significant interaction of and their effect on causing circRNA–miRNA HCC. In the present study, we have included all 15 studies reporting on the interaction of circRNA–miRNA and their possible role in inducing HCC (Figure 6).

Circ-ZNF652 is involved in the proliferation, glucose metabolism, invasion and migration of HCC cells [127]. Their expression was reported to be elevated in HCC patients. Circ-ZNF652 is involved in sponging the miR-29a-3p expression, whereas the miR-29a-3p targets the *guanylyl cyclase domain containing 1* (*GUCD1*) gene [128]. The *GUCD1* gene plays an important role in tumorigenesis and regeneration of the liver [129]. The overexpression of *GUCD1* reversed the effect of miR-29a-3p on the growth of HCC cells. The knockdown of circ-ZNF652 expression can prevent HCC cells from proliferation, invasion, migration and glucose metabolism [128].

CircFAT1 level strongly correlates with tumor size and TNM stage and is overexpressed in HCC cells and tissues [130]. CircFAT1 sponges miR-30a-5p expression. MiR-30a-5p targets the receptor expression-enhancing protein *3* (REEP3) protein. The inhibition of circFAT1 expression decreases in-vivo carcinogenesis, HCC cell proliferation and invasion. However, these actions are reversed by REEP3 overexpression. CircFAT1 sponges miR-30a-5p, controls REEP3 expression and leads to HCC progression [131].

Has_circ_104348 behaves as a ceRNA and is involved in the progression of HCC. It was reported that hsa_circRNA_104348 was significantly high in HCC tissue and it decreased cell apoptosis while promoting the cell invasion, migration and proliferation of HCC [132]. Hsa_circRNA_104348 specifically targets miR-187-3p and alters HCC cell proliferation, migration, invasion, and apoptosis, whereas miR-187-3p targets Rhotekin 2 (RTKN2) [133]. RTKN2 belongs to the class of certain proteins having a Rho-binding domain [134]. Has_circ_104348 target the miR-187-3p to control RTKN2 expression and stimulates the Wnt/-catenin pathway [133].

It was reported that has_circ_0000517 and SMAD family member 6 (SMAD6) were up-regulated while miR-326 was down-regulated in HCC tissues and cells. Hsa_circ_0000517 functions as a competitive endogenous RNA (ceRNA) for miR-326, controlling SMAD6 expression [135]. SMAD6 is a vital feedback suppressive modulator of bone morphogenetic protein (BMP)/SMAD signaling [136]. The downregulation of has_circ_0000517 in HCC cells inhibits cell division, migration and invasion, as well as causes cell cycle arrest in vitro and limits tumor growth in vivo [137]. miR-326 inhibitors overcame the restrictive effect of has_circ_0000517 knockdown on the malignant tendencies of HCC cells. Furthermore, the inhibition of miR-326 mimics’ effects on the malignant behaviors of HCC cells was reversed by SMAD6 upregulation [135].

Hsa_circ_0005397 promotes cell proliferation, apoptosis and metastasis. It was reported that has_circ_0005397 was upregulated in HCC tissues and cells. Has_circ_0005397 directly targets the miR-326 and sponge miR-326 expression [138], whereas miR-326 directly targets the *pyruvate dehydrogenase kinase 2* (*PDK2*) gene. PDK2 belongs to the PDK family and plays a crucial role in the progression of HCC (Hu, et al., 2017). *PDK2* overexpression reduced the anti-cancer effects of miR-326 in HCC. By sponging miR-326, has_circ_0005397 controls the expression of *PDK2*. Additionally, has_circ_0005397 down-regulation reduces tumor growth by lowering *PDK2* and increasing miR-326 [138].

CircASAP1 (hsa_circ_0085616) is derived from exons 2 and 3 of the *ASAP1* gene [139]. It was reported that CircASAP1 was upregulated in the HCC cell lines [140]. CircASAP1 competes with the HCC tumor suppressor miR-326 in the body. CircASAP1 targets the miR-326 and miR-326 targets the mitogen-activated protein kinase (MAPK1) and colony stimulating factor-1 (CSF-1 proteins) [141]. CircASAP1 controls the miR-326/MAPK1 signaling pathway, which promotes HCC cell proliferation and invasion. CircASAP1 controls the miR-326/CSF-1 pathway, which facilitates tumor-associated macrophage infiltration [139].

CircPTN promotes tumor growth in HCC. It was reported that circPTN overexpressed in the HCC cell line and tissues [142]. CircPTN interacts and sponges miR-326, whereas miR-326 targets and promotes epidermal growth factor receptor/phosphoinositol-3-kinases (ErbB/PI3K) expression [143]. ErbB binds with the large family of ligands and plays a crucial role in different pathways such as the defense mechanism of the liver during acute injury of the liver tissue [144]. PI3K controls different main cellular processes such as motility, proliferation, metabolism, growth and survival. Dysregulation of the PI3K signal transduction pathway involved in different malignancies (including HCC [145]). MiR-326 upregulation removes the inhibition of cell proliferation and reduces ErbB/PI3K expression in HCC cells via circPTN. CircPTN can stimulate the tumor growth of HCC according to loss-and-gain-of-function assays. The miR-326’s effects on HCC are absent when circPTN binding sites are mutated [143].

CircSLC7A11 (hsa_circ_0070975) precipitates in HCC growth and metastasis. It was reported that CircSLC7A11 is overexpressed in HCC cells and tissues. CircSLC7A11 masks the miR-330-3p expression, whereas miR-326 regulates cyclin dependent kinase 1 (CDK1) [146]. CDK1 is a regulatory checkpoint of the cell cycle that contributes to the regulation of cell proliferation and transcription processes [147]. The inhibitory effect of miR-330-3p on CDK1 was mitigated by circSLC7A11, precipitating the growth and metastasis of HCC [146].

Circ_0008450 is linked with TNM stage, tumor size, distant metastasis and lymphatic metastasis in HCC patients. It was reported that circ_0008450 expression increased in HCC tissues [148]. Circ_0008450 sponges the miR-431 expression, whereas miR-431 targets A-kinase anchor protein 1 (AKAP1) [149]. AKAP1 involves in mTOR pathway regulation and cancer growth [150]. Circ_0008450 controls AKAP1 expression by sponging miR-431. The downregulating of AKAP1 via miR-431 and circ_0008450 is involved in HCC development [149].

Circ-TCF4.85 promotes tumorigenicity but blocks the apoptosis in HCC cells. It was reported that circ-TCF4.85 is significantly upregulated in HCC tissues. Circ-TCF4.85 binds with miR-486-5p and positively regulates the expression of the *ATP-binding cassette subfamily F member 2* (*ABCF2*) gene [151]. ABCF2 belongs ATP-binding cassette (ABC) transporter superfamily, which facilitates the transport of particular molecules across lipid membranes [152]. Circ-TCF4.85 knockdown in HCC exhibited the ability to reverse the tumor-suppressive effects. The downregulation of *ABCF2* by miR-486-5p prevents HCC progression [151].

Circ-0091579 promotes cell proliferation, invasion and migration of HCC cells. It was reported that the expression of circ-0091579 is significantly increased in HCC tissues. Circ-0091579 combatively bind with miR-490-5p, whereas miR-490-5p directly binds with Cancer susceptibility candidate 3 (CASC3). CASC3 (also known as metastatic lymph node 51 (MLN51)) is involved in different types of cancer [153]. Circ-0091579 increased CASC3 via sponging miR-490-5p. Circ-0091579 and CASC3 silencing reduced HCC cell proliferation, migration, invasion and glycolysis [154].

Hsa_circ_0103809 promotes migration and proliferation, as well as blocks apoptosis. It was reported that hsa_circ_0103809 is overexpressed in the HCC cell line and tissues [155]. Hsa_circ_0103809 directly targets miR-490-5p and miR-490-5p is considered a potential tumor suppressor [156]. miR-490-5p targets the 3’-UTR of *sex-determining region Y-box 2* (*SOX2*) mRNA. *SOX2* acts as an oncogene in HCC pathogenesis [157]. Loss of function for hsa_circ_0103809 may result in decreased SOX2 expression and increased miR-490-5p expression. Hsa_circ_0103809 may promote the development of HCC malignancy by controlling the miR-490-5p/SOX2 signaling pathway [155].

Hsa_circ_0101432 (has_circ_RPPH1) promotes tumor growth. It was reported that hsa_circ_0101432 is upregulated in HCC. Hsa_circ_0101432 sponges miR-622 expression, whereas miR-622 targets the mitogen-activated protein kinase 1 (MAPK1) [158]. MAPK1 play an important role in different cellular pathways such as cell differentiation, proliferation, migration, growth and apoptosis [159]. Hsa_circ_0101432 increases cell proliferation, invasiveness, and tumor development in HCC cells via MAPK1 expression [158].

Circ_0061395 (CircBACH1) is involved in HCC progression through the inhibition of *p27* gene expression [160]. In HCC, circ_0061395 and *phosphoinositide-3-kinase regulatory subunit 3 (PIK3R3*) were upregulated but miR-877-5p was downregulated. Circ_0061395 competitively binds with miR-877-5p, whereas miR-877-5p targets the *PIK3R3* [161]. It has been proven that *PIK3R3* acts as an oncogene in HCC pathogenesis [162]. The knockdown of circ-0061395 reduced tumor development in-vivo and caused cell cycle arrest and apoptosis, as well as inhibited HCC cell invasion, migration and proliferation in vitro. The influence of circ_0061395 knockdowns on the malignant behaviors of HCC cells was reversed by the miR-877-5p inhibitor. The inhibitory effect of the miR-877-5p mimic on the malignant tendencies of HCC cells was reversed by the overexpression of *PIK3R3* [161].

Circ-BIRC6 acts as an oncogene in HCC. It was reported that circ-BIRC6 is upregulated in HCC tissues and cells [142]. Circ-BIRC6 works as a molecular sponge for miR-877-5p. However, MiR-877-5p directly targets tyrosine 3-monooxygenase/tryptophan 5-monooxygenase activation protein zeta (YWHAZ) [163]. In the case of HCC, YWHAZ also acts as an oncogenic protein [164]. In HCC tissues and cells, miR-877-5p was downregulated while circ-BIRC6 and YWHAZ were increased. The knockdown of miR-877-5p reversed the inhibitory effects of circ-BIRC6 depletion on the development of HCC tumors. [163].

**Figure 7:**
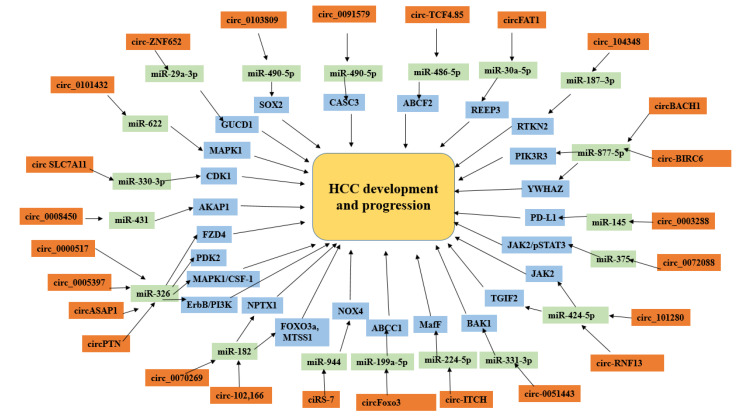
CircRNA–miRNA interactions in HCC development and progression.

In data mining, we observed almost all possible interactions of selected miRNAs with their target circRNAs. The downstream proteins involved in HBV, HCV or alcohol-induced HCC development and progression (Supplementary Table S3) reported by different experimental studies. We investigated and generated an insilico model of these circRNAs–miRNAs and their downstream protein interaction in HCC development and progression, all of these interactions has been summarized in Figure 7.

## 4. Discussion

HCC is the most prevalent type of liver cancer and the main cause of death worldwide. There are different factors involved in HCC development (such as HBV, HCV, alcoholism and many others [165]). Various precancerous lesions such as liver fibrosis and cirrhosis are involved in HCC development; these two conditions have also been extensively studied, but their detailed mechanism has not been discovered yet [166]. The diagnostic methods and therapeutic potential have constantly been upgraded, but there are still several patients with HCC who are discovered at the final stage with acute liver dysfunction. Therefore, finding new diagnostic and prognostic targets for HCC treatment is very important [165].

In previous years, a large amount of data reported the regulatory roles of miRNA in hepatic carcinogenesis [167] and reveals their association with several risk factors such as HCV, HBV infection, autoimmune liver disease, drug toxicity and alcohol consumption [168]. It targets genes that participate in DNA repair, proliferation, tumorigenesis, invasion, metastasis and apoptosis [169]. In the process of HCV replication, multiple miRNAs are also involved. Moreover, the downregulation of miRNA processing machinery genes (including *Dicer*, *Drosha*, *Ago2*, *TRBP* and *DGCR8*) has been observed to decrease the synthesis of mature miRNA, leading to HCC development [59]. For instance, reduced miR-16 and miR-199a expression levels and enhanced miR-155 and miR-122 expression levels were linked with HCV-induced HCC and were thought to be reliable, non-invasive biomarkers of HCC. In cell line and animal models, HCC development caused by mi-484 was found by Yang et al. (2016) [170].

In recent studies, scientists have also observed the activity of circRNAs in various cancers [171]. Currently, the broad distribution and functional control of circRNAs in human malignancies has been extensively validated [172]. A group of circRNAs have been identified though RNA-seq and are proven to be common in hepatocellular, colorectal and lung cancer, which offers promising molecular biomarkers and therapeutic targets for several human diseases [173]. A huge amount of data suggest that circRNAs may act as ceRNAs or miRNA sponges to facilitate or suppress tumor growth. For instance, circRNA-100290 co-expressed with CDK6 as a miR-29 sponge and contributed to the tumorigenesis of oral cancer [171]. Few researchers have investigated the link between circRNAs and cancer. The existing methods to identify and characterize circRNAs are still not confirmed and are also debatable in effectiveness. The emerging roles of the ceRNA complex network that communicate with miRNA and the vast majority of circRNA structures that have been found are playing important roles in cellular regulation and human disease, eliciting fascinating new research directions [174]. circRNAs comprise one or more miRNA response elements (MREs), which work as miRNA sponges to negatively regulate miRNA expression. Numerous studies indicate that circRNAs (through sponging miRNAs) control the gene expression and take part in a variety of biological activities, reducing the inhibitory effect of miRNA on their target genes. The circRNA–miRNA–mRNA interaction has been stated to be linked with cancer progression. Due to their high degree of stability, specific expression patterns and distinctive structure, circRNAs are considered to be potential biomarkers against various diseases [172].

In the present study, we have investigated the miRNA expression in HCC induced by HBV, HCV and alcohol through literature mining. This study is based on four groups of literature reporting various HCC aetiologies and their association with aberrant miRNA expression. Most of the included studies reported the role of miRNA in HCC development as being due to unknown aetiology (45 studies), while other studies revealed the role of miRNA in HCC as being due to HBV infection (18 studies). Some studies linked the close association of HCV-induced HCC and miRNA (9 studies), while only a few studies related their association to alcoholism (4 studies). In the present study, only 88 miRNAs were prioritized based on their significant expression level (*p* ˂ 0.05) in HCC patients reported in the selected studies. Among these screened miRNAs, 47 miRNAs were related to HCC (with unknown aetiology), 24 miRNAs were related to HBV-induced HCC, 15 miRNAs were related to HCV-induced HCC and 6 miRNAs were related to alcoholism (alcoholic related hepatitis). Additionally, we have also identified common miRNAs associated with HCC development by various causative agents (HCV, HBV and alcohol) (Figure 3). miR-223 was found to commonly associate with HCC development due to all causative agents. Eventually, several significant upregulated and downregulated miRNA signatures were identified that have played significant roles in HCC tumorigenesis (Table 1). In addition to the role of miRNA in HCC pathogenesis, circRNA also played a critical role in HCC development by regulating the miRNAs’ expression. Therefore, we also investigated dysregulated circRNAs in HCC tissues from the worldwide studies. Moreover, we have screened out different circRNAs that target the miRNAs and act as a sponge, downregulate or upregulate the miRNAs’ expression by targeting different miRNAs and their downstream pathways and play significant roles in HCC progression.

Different studies reported the miRNAs and circRNAs and their role in HCC development. Comprehensive studies on circRNA–miRNA interactions and their significant role in HBV, HCV and alcohol-induced HCC development and progression is lacking. Therefore, in the present study, we prioritized dysregulated circRNAs and miRNAs and their possible interactions in HBV, HCV and alcohol-induced HCC (Figure 5).

We have found that has_circ_101280 and miR-375 interaction play a critical role in HCC development. Has_circ_101280 overexpression increased proliferation and decreased apoptosis via miR-375 downregulation in HCC cells. In the nude mice model, the silencing of has_circ_101280 resulted in the overexpression of miR-375 and the downregulation of JAK2, which together prevented the formation of HCC tumors (Figure 6) [111]. circ-TCF4.85 and miR-486-5p interaction also play a significant role in HCC progression. circ-TCF4.85 promotes HCC development and progression through the direct regulation of miR-486-5p and the indirect regulation of *ABCF2* expression. The knockdown of circ-TCF4.85 expression increases the miR-486-5p expression, inhibits cell proliferation, migration and invasion abilities, and induces apoptosis via suppression of *ABCF2* in HCC (Figure 6) [151].

Exosomal circ-0051443 also controls BAK1 and prevents HCC progression by acting as a miR-331-3p sponge. circ-0051443 serves as an intercellular communication regulator in HCC carcinogenesis (Figure 6) [114]. Hsa_circ_0005397 targets miR-326 and indirectly controls PDK2 expression, thus affecting HCC development. The upregulation of PDK2 in HCC cell lines declined the anti-cancer roles of miR-326. These findings revealed that, through upregulating PDK2, has_circ_0005397 acted as a molecular sponge for miR-326, thus promoting the progression of HCC (Figure 6) [138]. miR-944 acts as a tumor suppressor in HCC cancer, and its expression is controlled by ciR-7. MiR-944 targets the NOX4 in HCC cells. NOX4 is a member of the NAPDH oxidases family and is crucial for the generation of ROS. ciR-7 regulated HCC development by causing the formation of ROS (Figure 6) [122].

Has_circ_0070269 inhibited the HCC progression by targeting and controlling the miR-182/NPTX1 axis. miR-182 directly targets NPTX1 in HCC cells, and miR-182 mimics mitigated the impacts of has_circ_0070269 overexpression on NPTX1 expression in HCC cells (Figure 6) [116]. Hsa_circ_0003288 stimulated the invasion and EMT in HCC by negatively regulating miR-145 and positively regulating PD-L1 expression levels. The slicing of has_circ_0003288 expression decreased the levels of PD-L1 mRNA and protein in HCC. The inhibition of has_circ_0003288 expression in HCC decreased tumor development and EMT using in vivo animal models. EMT and tumor development are related to the PI3K/AKT signaling pathway (Figure 6) [107]. Therefore, in our study, we have identified all possible miRNAs related with HBV, HCV and alcohol-induced HCC. We also found 45 common miRNAs related to HCC with unknown aetiology. Though data mining, we have observed the critical role of various circRNA –miRNA interactions in HCC. In this study, we have reported these circRNA–miRNA interactions and their role in HCC development and progression. These circRNA–miRNA interactions can be used as prognostic biomarkers or therapeutic targets for the treatment of HCC. However, the main limitation of this study is the absence of in vivo validation. There is a need for further experimental validation to check these circRNA–miRNA interactions in HCC development and progression.

## 5. Conclusions

HCC related miRNA signatures that are significantly upregulated or downregulated are identified in this study. These miRNA signatures were used as diagnostic biomarkers and therapeutic targets for HCC. There is a need for further study to identify the mechanisms underlying HCC development through miRNA involvement. This is explained by the emerging role circRNA–miRNA interactions play in the molecular mechanisms and expression of genes involved in HCC development. By offering a new perspective of circRNA–miRNA interactions in HCC, this study aids in the discovery of new possible diagnostic and prognostic biomarkers.

## Figures and Tables

**Table 1:** miRNAs reported in HCC patients from different studies.

Sr. No	miRNAs	Region	References	Sr. No	miRNAs	Region	References
1	miR-10b	China	[11]	46	miR-222	China	[12]
2	miR-17-5p	Turkey	[13]	47	miR-223	USA	[14]
3	miR-18a	China	[15]		miR-223	China	[16]
4	miR-19a	China	[17]		miR-223	China	[17]
5	miR-21	India	[18]	48	miR-224	China	[19]
6	miR-22	China	[20]	49	miR-224-5p	China	[21]
7	miR-22-3p	China	[22]	50	miR-300	China	[23]
8	miR-23b	China	[16]	51	miR-320a	China	[24]
9	miR-23b-3p	China	[21]	52	miR-325	China	[25]
10	miR-24-3p	Turkey	[13]	5354	miR-326	Egypt	[26]
	miR-24-3p	China	[27]		miR-331-3p	China	[21]
11	miR-27a	Egypt	[28]	55	miR-330-3p	China	[29]
12	miR-28-5p	China	[30]	56	mi-345	China	[31]
13	miR-29a-3p	China	[32]	58	miR-361-5p	China	[33]
14	miR-30a-5p	China	[34]	57	miR-362-5p	China	[35]
15	miR-32-5p	China	[36]	58	miR-370	China	[37]
16	miR-34a	USA	[38]	59	miR-371a-5p	China	[39]
17	miR-34c	China	[40]	60	miR-373-3p	China	[41]
18	miR-93	USA	[42]	61	miR-375	China	[43]
19	miR-95-3p	China	[44]	62	miR-384	China	[45]
20	miR-96	China	[46]	63	miR-424-5p	China	[47]
21	miR-99a	China	[48]	64	miR-431	China	[49]
22	miR-122	USA	[50]	65	miR-452-3p	China	[51]
23	miR-122-5p	China	[21]	66	miR-486-5p	China	[52]
24	miR-125a	Egypt	[53]	67	miR-490-5p	China	[54]
25	miR-125b-5p	Germany	[55]	68	miR-491	China	[56]
26	miR-129-2	China	[57]	69	miR-493	China	[58]
27	miR-132	China	[59]	70	miR-494-3p	China	[21]
28	miR-133b	China	[60]	71	miR-503	China	[61]
29	miR-137	China	[62]	72	miR-548p	China	[63]
30	miR-139	Egypt	[53]	73	miR-603	China	[64]
31	miR-145	India	[18]	74	miR-622	China	[65]
32	miR-149	China	[66]	75	miR-744-5p	China	[67]
33	miR-150-5p	China	[68]	76	miR-801	China	[16]
34	miR-152	France	[69]	77	miR-877-5p	China	[70]
35	miR-155	China	[71]	78	miR-922	China	[72]
36	miR-182	Egypt	[73]	79	miR-940	China	[74]
37	miR-187-3p	China	[75]	80	miR-944	USA	[14]
38	miR-192	China	[16]	81	miR-1236	China	[76]
39	miR-193-5p	India	[77]	82	miR-1287	China	[78]
40	miR-194	China	[16]	83	miR-1296	China	[79]
41	miR-195	Egypt	[80]	84	miR-1468	China	[72]
42	miR-199a-5p	Germany	[81]	85	miR-3194-3p	China	[56]
43	miR-205	China	[82]	86	miR-3651	China	[83]
44	miR-210-3p	Japan	[59]	87	miR-4319	China	[84]
45	miR-211	China	[85]	88	miR-6875-3p	China	[86]

**Table 3:** miRNA expression in HBV-induced HCC.

Scheme	Symbol	Expression	*p*-Value	References	Sr. No	Symbol	Expression	*p*-Value	References
1	miR-18a	High	<0.01	[15]	13	miR-194	High	<0.01	[16]
2	miR-19a	High	˂0.05	[17]	14	miR-205	Low	<0.01	[82]
3	miR-21	Low	0.0487	[18]	15	miR-210-3p	High	<0.0308	[59]
4	miR-22	High	0.027	[20]	16	miR-222	High	0.046	[12]
5	miR-23b	High	<0.01	[16]	17	miR-223	Low	<0.01	[16]
6	miR-24-3p	High	˂0.05	[27]	18	miR-224	High	˂0.05	[19]
7	miR-34c	Low	<0.01	[40]	19	miR-371a-5p	High	<0.05	[39]
8	miR-96	High	˂0.01	[46]	20	miR-375	Low	˂0.05	[43]
9	miR-132	Low	0.021	[59]	21	miR-384	Low	<0.05	[45]
10	miR-145	Low	0.0486	[18]	22	miR-548p	Low	<0.01	[63]
11	miR-192	High	<0.01	[16]	23	miR-801	High	<0.01	[16]
12	miR-193a-5p	Low	<0.03	[77]	24	miR-1236	Low	<0.05	[76]

**Table 4:** miRNA expression in HCV-induced HCC.

Sr. No	Symbol	Expression	*p*-Value	References	Sr. No	Symbol	Expression	*p*-Value	References
1	miR-17-5p	Low	˂0.05	[13]	9	miR-155	High	˂0.05	[71]
2	miR-23b-3p	Low	0.038	[21]	10	miR-182	Low	0.015	[73]
3	miR-24-3p	Low	˂0.05	[13]	11	miR-195	Low	0.04	[80]
4	miR-27a	High	0.03	[28]	12	miR-223	Low	˂0.05	[17]
5	miR-122-5p	High	0.011	[21]	13	miR-224-5p	High	0.018	[21]
6	miR-125a	Low	<0.01	[53]	14	miR-331-3p	High	0.020	[21]
7	miR-139	Low	<0.01	[53]	15	miR-494-3p	High	0.025	[21]
8	miR-152	Low	<0.01	[69]					

**Table 5:** miRNA expression in alcohol-induced HCC.

Sr. No	Symbol	Expression in HCC	*p*-Value	References
1	miR-22-3p	High	˂ 0.01	[22]
2	miR-122	High	˂ 0.05	[50]
3	miR-125b-5p	Low	0.019	[81]
4	199a-5p	Low	0.04	[81]
5	miR-223	High	0.037	[14]
6	miR-944	High	0.019	[14]

**Table 6:** miRNA expression in HCC.

Sr. No	Symbol	Expression	*p*-Value	References	Sr. No	Symbol	Expression	*p*-Value	References
1	miR-10b	High	< 0.01	[11]	25	miR-362-5p	High	< 0.01	[35]
2	miR-28-5p	Low	<0.05	[30]	26	miR-370	Low	<0.01	[37]
3	miR-29a-3p	Low	<0.05	[32]	27	miR-373-3p	Low	<0.05	[41]
4	miR-30a-5p	Low	<0.05	[34]	28	miR-424-5p	Low	<0.05	[47]
5	miR-32-5p	High	<0.01	[36]	29	miR-431	Low	<0.05	[49]
6	miR-34a	High	<0.01	[38]	30	miR-452-3p	High	<0.01	[51]
7	miR-93	High	0.022	[42]	31	miR-486-5p	Low	<0.05	[52]
8	miR-95-3p	High	<0.01	[44]	32	miR-490-5p	Low	<0.01	[54]
9	miR-99a	Low	<0.01	[48]	33	miR-491	High	<0.01	[56]
10	miR-129-2	Low	<0.01	[57]	34	miR-493	Low	<0.05	[58]
12	miR-133b	Low	<0.01	[60]	35	miR-503	Low	<0.01	[61]
13	miR-137	Low	<0.05	[62]	36	miR-603	High	<0.01	[64]
14	miR-149	Low	0.023	[66]	37	miR-622	Low	<0.01	[65]
15	miR-150-5p	Low	0.015	[68]	38	miR-744-5p	Low	<0.01	[67]
16	miR-187-3p	Low	<0.01	[75]	39	miR-877-5p	Low	<0.01	[70]
17	miR-211	Low	<0.01	[85]	40	miR-922	High	<0.05	[72]
18	miR-300	Low	0.02	[23]	41	miR-940	Low	<0.05	[74]
19	miR-320a	Low	<0.05	[24]	42	miR-1296	Low	<0.05	[79]
20	miR-325	Low	<0.05	[25]	43	miR-1468	High	<0.05	[72]
21	miR-326	Low	<0.05	[100]	44	miR-3194-3p	Low	<0.05	[56]
22	miR-330-3p	High	<0.01	[29]	45	miR-3651	High	<0.01	[83]
23	mi-345	Low	<0.05	[31]	46	miR-4319	Low	<0.01	[84]
24	miR-361-5p	Low	<0.01	[33]	47	miR-6875-3p	High	<0.05	[86]

## Data Availability

The data presented in this study are available upon request from the corresponding author. The information regarding the miRNAs reported in study is available in Table S1, in the Supplementary Materials section of this manuscript.

## Supplementary Materials

The following supporting information can be downloaded at: https://www.mdpi.com/article/10.3390/genes14010013/s1. Table S1: miRNAs related with HBV, HCV and alcohol induced HCC; Table S2: Exosomal miRNAs expression in HCC development; Table S3: CircRNAs-miRNAs interactions in HCC.
