# Supplementary material for: The Role of miRNAs, circRNAs and Their Interactions in Development and Progression of Hepatocellular Carcinoma: An Insilico Approach
Source: Genes (Basel). 2022 Dec 21;14(1):13. doi: 10.3390/genes14010013 (PMC9858589; doi:10.3390/genes14010013)
Supplement: Supplementary file 1 [file genes-14-00013-s001.zip › genes-2052089-supplementary.pdf]

# Supplementary Materials

**Table S1.** miRNAs expression in HCC development

| Sr. No | miRNAs | Expression | P-value | Reported region | References |
|---|---|---|---|---|---|
| 1 | miR-10b | High | < 0.01 | China | (Dongliang Li et al., 2018) |
| 2 | miR-17-5p | Low | < 0.05 | Turkey | (Oksuz et al., 2015) |
| 3 | miR-18a | High | < 0.01 | China | (L. Li et al., 2012) |
| 4 | miR-19a | High | < 0.01 | China | (G. Yu et al., 2016) |
| 5 | miR-21 | Low | 0.0487 | India | (Bandopadhyay et al., 2014) |
| 6 | miR-22 | High | 0.027 | China | (Jiang et al., 2011) |
| 7 | miR-22-3p | High | < 0.01 | China | (D. Chen et al., 2021) |
| 8 | miR-23b | High | < 0.01 | China | (J. Zhou et al., 2011) |
| 9 | miR-23b-3p | High | < 0.01 | China | (J. Li et al., 2019) |
| 10 | miR-24-3p | Low | < 0.05 | Turkey | (Oksuz et al., 2015) |
| | miR-24-3p | High | < 0.05 | China | (Meng, Wang, & Jia, 2014) |
| 11 | miR-27a | High | 0.03 | Egypt | (Rashad, El-Shal, Shalaby, & Mohamed, 2018) |
| 12 | miR-28-5p | Low | < 0.05 | China | (Shi, Teng, & Biochemistry, 2015) |
| 13 | miR-29a-3p | Low | < 0.05 | China | (Z. Xiao, Wang, Ding, & bioscience, 2019) |
| 14 | miR-30a-5p | Low | < 0.05 | China | (LiWF, 2016) |
| 15 | miR-32-5p | High | < 0.01 | China | (Fu et al., 2018) |
| 16 | miR-34a | High | < 0.01 | USA | (N. Li et al., 2009) |
| 17 | miR-34c | Low | < 0.01 | China | (Y. Wang et al., 2015) |
| 18 | miR-93 | High | 0.022 | USA | (Ohta et al., 2015) |
| 19 | miR-95-3p | High | < 0.01 | China | (Ye et al., 2016) |
| 20 | miR-96 | High | < 0.01 | China | (Y. Chen, Dong, Yu, & Wang, 2015) |

| | | | | | |
|---|---|---|---|---|---|
| 21 | miR-99a | Low | < 0.01 | China | (Dong Li et al., 2011) |
| 22 | miR-122 | High | < 0.05 | USA | (Ambade, Satishchandran, & Szabo, 2016) |
| 23 | miR-122-5p | High | 0.011 | China | (J. Li et al., 2019) |
| 24 | miR-125a | Low | < 0.01 | Egypt | (El-Ahwany et al., 2019) |
| 25 | miR-125b-5p | Low | 0.019 | Germany | (Felgendreff et al., 2020a) |
| 26 | miR-129-2 | Low | < 0.01 | China | (Liu et al., 2016) |
| 27 | miR-132 | Low | 0.021 | China | (Morishita et al., 2020) |
| 28 | miR-133b | Low | < 0.01 | China | (Hui Li, Xiang, Liu, Xu, & Tang, 2017) |
| 29 | miR-137 | Low | < 0.05 | China | (J. Wang et al., 2021) |
| 30 | miR-139 | Low | < 0.01 | Egypt | (El-Ahwany et al., 2019) |
| 31 | miR-145 | Low | 0.0486 | India | (Bandopadhyay et al., 2014) |
| 32 | miR-149 | Low | 0.023 | China | (Luo et al., 2015) |
| 33 | miR-150-5p | Low | 0.015 | China | (T. Li et al., 2014) |
| 34 | miR-152 | Low | < 0.01 | France | (Miquelestorena - Standley et al., 2018) |
| 35 | miR-155 | High | < 0.05 | China | (Y. Zhang et al., 2012) |
| 36 | miR-182 | Low | 0.015 | Egypt | (Shaheen et al., 2018) |
| 37 | miR-187-3p | Low | < 0.01 | China | (Dou et al., 2016) |
| 38 | miR-192 | High | < 0.01 | China | (J. Zhou et al., 2011) |
| 39 | miR-193-5p | Low | < 0.03 | India | (Ghosh et al., 2016) |
| 40 | miR-194 | High | < 0.01 | China | (J. Zhou et al., 2011) |
| 41 | miR-195 | Low | 0.04 | Egypt | (Motawi, Shaker, El-Maraghy, & Senousy, 2015) |
| 42 | miR-199a-5p | Low | 0.04 | Germany | (Felgendreff et al., 2020b) |
| 43 | miR-205 | Low | < 0.01 | China | (T. Zhang et al., 2013) |

| | | | | | |
|---|---|---|---|---|---|
| 44 | miR-210-3p | High | < 0.0308 | Japan | (Morishita et al., 2020) |
| 45 | miR-211 | Low | < 0.01 | China | (Deng et al., 2016) |
| 46 | miR-222 | High | 0.046 | China | (Qi et al., 2011) |
| 47 | miR-223 | High | 0.037 | USA | (Zheng et al., 2017) |
| | miR-223 | Low | < 0.01 | China | (J. Zhou et al., 2011) |
| | miR-223 | Low | < 0.05 | China | (G. Yu et al., 2016) |
| 48 | miR-224 | High | < 0.05 | China | (H. Zhang, Chen, Yang, Zhang, & Wang, 2014) |
| 49 | miR-224-5p | High | 0.018 | China | (J. Li et al., 2019) |
| 50 | miR-300 | Low | 0.02 | China | (R. Wang et al., 2018) |
| 51 | miR-320a | Low | < 0.05 | China | (Lv et al., 2017) |
| 52 | miR-325 | Low | < 0.05 | China | (Huifen Li, Huang, & Luo, 2015) |
| 53 | miR-326 | Low | < 0.05 | China | (S. Hu, Ran, Chen, Zhang, & Xu, 2017) |
| 54 | miR-330-3p | High | < 0.01 | China | (J. Li et al., 2019) |
| 55 | miR-331-3p | High | 0.02 | China | (X Zhao et al., 2019) |
| 56 | mi-345 | Low | < 0.05 | China | (M. Yu et al., 2017) |
| 57 | miR-361-5p | Low | < 0.01 | China | (Cheng et al., 2019) |
| 58 | miR-362-5p | High | < 0.01 | China | (Ni et al., 2015) |
| 59 | miR-370 | Low | < 0.01 | China | (He & He, 2019) |
| 60 | miR-371a-5p | Low | < 0.05 | China | (P.-s. Bai, Hou, & Kong, 2018) |
| 61 | miR-373-3p | High | < 0.05 | China | (Hongbin Li et al., 2022) |
| 62 | miR-375 | Low | < 0.05 | China | (W. Zhang et al., 2020) |
| 63 | miR-384 | Low | < 0.05 | China | (P. s. Bai, Xia, Sun, Kong, & Medicine, 2017) |
| 64 | miR-424-5p | Low | < 0.05 | China | (Du et al., 2019) |
| 65 | miR-431 | Low | < 0.05 | China | (Sun et al., 2015) |

| | | | | | |
|---|---|---|---|---|---|
| 66 | miR-452-3p | High | < 0.01 | China | (Tang et al., 2017) |
| 67 | miR-486-5p | Low | < 0.05 | China | (X. P. Huang et al., 2015) |
| 68 | miR-490-5p | Low | < 0.01 | China | (B. Xu et al., 2017) |
| 69 | miR-491 | High | < 0.01 | China | (Y. Zhou et al., 2013) |
| 70 | miR-493 | Low | < 0.05 | China | (W. Ding et al., 2018) |
| 71 | miR-494-3p | High | 0.025 | China | (J. Li et al., 2019) |
| 72 | miR-503 | Low | < 0.01 | China | (Y. Xiao et al., 2016) |
| 73 | miR-548p | Low | < 0.01 | China | (X. M. Hu et al., 2016) |
| 74 | miR-603 | High | < 0.01 | China | (Lin et al., 2021) |
| 75 | miR-622 | Low | < 0.01 | China | (Song et al., 2015) |
| 76 | miR-744-5p | Low | < 0.01 | China | (W. Huang et al., 2021) |
| 77 | miR-801 | High | < 0.01 | China | (J. Zhou et al., 2011) |
| 78 | miR-877-5p | Low | < 0.01 | China | (Yan, Qiu, Sun, & Li, 2018) |
| 79 | miR-922 | High | < 0.05 | China | (Liu et al., 2018) |
| 80 | miR-940 | Low | < 0.05 | China | (D. Ding et al., 2016) |
| 81 | miR-944 | High | 0.019 | USA | (Zheng et al., 2017) |
| 82 | miR-1236 | Low | < 0.05 | China | (C. Zhang, Liu, & Zhang, 2020) |
| 83 | miR-1296 | Low | < 0.05 | China | (Q. Xu et al., 2017) |
| 84 | miR-1468 | High | < 0.05 | China | (Liu et al., 2018) |
| 85 | miR-3194-3p | Low | < 0.05 | China | (Yao et al., 2019) |
| 86 | miR-3651 | High | < 0.01 | China | (Xinyang Zhao, Song, Miao, Zhu, & therapy, 2019) |
| 87 | miR-4319 | Low | < 0.01 | China | (Han et al., 2019) |
| 88 | miR-6875-3p | High | < 0.05 | China | (Xie et al., 2019) |

**Table S2.** miRNAs related with HBV, HCV and alcohol induced HCC.

| Sr. No | miRNAs | Expression in serum of HCC | Region | References |
|---|---|---|---|---|
| **1** | Exosomal miR-10b | Upregulated | China | Liu, W.H., Ren, L.N., et al 2015 |
| **2** | Exosomal miR-18a | Upregulated | Korea | Sohn, W., et al 2015. |
| **3** | Exosomal miR-21 | Upregulated | China | Liu, W., Chen, S. and Liu, B., 2016 |
| **4** | Exosomal miR-34a | Downregulated | China | Jiao, C., Jiao, X., et al 2017 |
| **5** | Exosomal miR-34c | Downregulated | China | Jiao, C., Jiao, X., et al 2017 |
| **6** | Exosomal miR-93 | Upregulated | China | Xue, X., et al., 2018. |
| **7** | Exosomal miR-122 | Downregulated | USA | Fan, B., et al 2015. |
| **8** | Exosomal miR-195 | Downregulated | Korea | Sohn, W., et al 2015. |
| **9** | Exosomal miR-222 | Upregulated | Korea | Sohn, W., et al 2015. |
| **10** | Exosomal miR-223 | Upregulated | UK | Aucher, A., et al 2013. |
| **11** | | | | |
| 12 | Exosomal miR-224 | Upregulated | Korea | Sohn, W., et al 2015. |

**Table S3.** CircRNAs-miRNAs interactions in HCC.

| circRNAs | miRNA | Pathology | Target gene, protein | Sample | Methodology | References |
|---|---|---|---|---|---|---|
| hsa_circ_0000517 | competed miR-326 | HCC | SMAD6, IGF1R | tissue and cell line | cell culture, RT-PCR, flow cytometry, RIP, westren blot, different assays | Shuwei He, et al 2020 DOI: 10.1186/s12935-020-01447-w |
| hsa_circ_0005075 | target and inhibit miR-431 | HCC | Bcl-D | tissue | cell culture, western blot, RNA extraction, RT-PCR, different assays | Ming-Fang L., et al 2018 DOI: 10.1016/j.biopha.2018.01.150 |
| hsa_circ_0005397 | regulating the miR-326 | HCC | PDK2 | tissue | cell culture, RNA extration, RT-PCR, Caspase-3 activity assay, | Jianzhuang Gong, et al 2021 doi.org/10.1002/jgm.3332 |
| circ_0008450 | sponge miR-548p | HBV induced HCC | HKa | tissue and cell line | RNA extraction, RT-PCR, different assay | Juan Zhang, et al 2018 doi.org/10.1002/jcb.28224 |
| | sponging miR-431 | HCC | AKAP1 via | cell and tissue | RT-PCR, Cell Counting Kit-8, flow cytometry and glycolysis assays, westren blot | Du Q, Han et al 2020. doi: 10.3892/ol.2020.12251. |
| Circ-0051443 | competitive bound to miR-331-3p | HCV induced HCC | BAK1 | tissue and cell line | cell culture, exsome isolation, westren blot, RT-PCR, IHC, RIP, different assays | WeiChena, et al 2020 doi.org/10.1016/j.canlet.2020.01.022 |
| circ_0061395 | negitively regulating the miR-877-5p | HCC | PIK3R3 | tissue, cell line, serum | cell culture, TEM, westren blot, different assyas, RT-PCR, flow cytometry, RIP, IHC | Yanhui Yu, et al 2021 DOI: 10.1186/s12935-020-01695-w |
| hsa_circ_0070269 | sponging miR-182 | HCV induced HCC | NPTX1 | tissue and cell line | ell culture, RT-PCR, different assay, westren blot | Xiaotong Su, et al 2019 DOI: 10.1016/j.biopha.2019.109497 |
| circ_0072088 | sponge of miR-375 | HBV induced HCC | JAK2 and p-STAT3 | tissuee and cell line | cell culture, RT-PCR, different assay, flow cytometry, westren blot, | Li et al., 2021DOI:10.1002/iub.2520 |
| hsa_circ_0085616 | miR-326 | HCC | MAPK1/CSF-1 | Tissue and cell line | CircRNA pull down assay, RNA extreaction, RNA immunoprecipetation (RIP), qRT-PCR, westren blot, ELISA | Zhi-Qiang Hu, et al 2020 DOI: 10.1002/hep.31068 |

| | | | | | | |
|---|---|---|---|---|---|---|
| circ_0091579 | interact miR-490-5p | HCC | CASC3 | tissue and cell line | cell culture, RNA extraction, RT-PCR, westren blot, different assays | Wei Liu, et al 2021 DOI: 10.1089/cbr.2019.3472 |
| hsa_circ_0101432 | absorbing miR-622 | HCC | MAPK1 | tissue | RNA extartion, microaaray, cell culture, RT-PCR | Haibo Zou, et al 2019 doi.org/10.1080/15384101.2019.1618120 |
| hsa_circ_0103809 | suppressed miR-490-5p | HCC | SOX2 | cell lines and tissue | cell culture, RT-PCR, Westren blot, IHC, flow cytometry, different assays | Huajie Cai, et al 2018 PMCID: PMC6038084 |
| hsa_circ_101280 | sponging miR-375 | HBV induced HCC | JAK2 | tissue | cell culture, RT-PCR, different assay, flow cytometry | Shuang Cao, et al 2018 doi.org/10.1111/imcb.12213 |
| circ-102,166 | sponge miR-182 | HCV induced HCC | FOXO3a, MTSS1, SOX7, p-RB and c-MYC | Tissue, cell line | RNA extraction, RT-PCR, | Rong Li, et al 2021 doi.org/10.1007/s13402-020-00564-y |
| circRNA_104348 | target miR-187–3p | HCC | RTKN2, Wnt/β-catenin signaling pathway | tissue | RNA extraction, RT-PCR, cell culture, different assay | Guanqun Huang, et al 2019 .doi.org/10.1038%2Fs41419-020-03276-1 |
| circ-BIRC6 | sponge of miR-877-5p | HCC | YWHAZ | cell and tissue | RT-PCR,flow cytometry, different assays | Liu Y, et al. 2020 doi: 10.2147/OTT.S261700. |
| circFAT1 | sponges miR-30a-5p | HCC | REEP3 | tissue and cell line | cell culture, RT-PCR, FISH, RIP, different assays, westren blot | Hailiang Wei, et al 2020 https://doi.org/10.1111/jcmm.16085 |
| circ-ITCH | sponge miR-224-5p | HCV induced HCC | MafF | tissue and cell line | cell culture, IHC, RNA extraction, RT-PCR, , different assay, westren blot | Minhua Wu, et al 2020 doi: 10.3727/096504020X15796890809840 |
| circPTN | sponging miR-326 | HCC | ErbB/PI3K | tissua and cell line | cell culture, RT-PCR, different assays, FISH | Benli Jia, et al 2020 doi: 10.2147/OTT.S251300 |
| circSLC7A11 | sponge miR-330-3p | HCC | CDK1 | tissue and cell line | cell culture, different assya, RT-PCR, IHC,RIP,Westrn blot | Yu Huang, et al 2021 https://doi.org/10.1186/s12935-021-02351-7 |
| circ-RNF13; | miR-424-5p spong | HBV induced HCC | TGIF2 | tissue and cell | cell culture, RT-PCR, different assays | Chen Y, et al 2020. doi: 10.17305/bjbms.2020.5266. . |
| circ-TCF4.85 | miR-486-5p knodown the cicRNA | HCC | ABCF2 | tissue and cell line | cell culture, RT-PCR, Microarray-based circRNA expression profiling, westren blot, flow cytometry, FISH, IHC, different assays | Jun Gao, et al 2019 https://doi.org/10.1002/1878-0261.12603 |
| Circ-ZNF652 | target miR-29a-3p | HCC | GUCD1 | serum and tissue | RT-PCR, westren blot,RNase R digestion assay, MTT, RIP, different assays, | Li Y, et al. 2020 doi: 10.2147/CMAR.S259424. |
| ciRS-7 | miR-944 | alcohol induced HCC | NOX4 Pathway | tissue and cell line | qRT-PCR), Western blot, RNA pull-down, and luciferase reporter assay | Chuangjie Mao, et al 2022 DOI: 10.1615/CritRevEuk |

| | | | | | | |
|---|---|---|---|---|---|---|
| | | | | | | aryotGeneExpr.2022 039225 |
| circFoxo3 | miR-199a-5p | alcohol induced HCC | ABCC1 | tissue and cell line | cell culture, RT-PCR, Westren blot, IHC, flow cytometry, different assays | Wei Huang et al 2020 DOI: 10.2147/OTT.S243571 |

**References**


1. Li, Dongliang, Yongjian Zhang, He Zhang, Chao Zhan, Xin Li, Tu Ba, Zini Qiu, Guixiang Lv, Chendan Zou, Chuxuan %J Journal of Experimental Wang, and Clinical Cancer Research. "Cadm2, as a New Target of Mir-10b, Promotes Tumor Metastasis through Fak/Akt Pathway in Hepatocellular Carcinoma." 37, no. 1 (2018): 1-11.
2. Qi, Peng, Shu-qun Cheng, Hao Wang, Nan Li, Yue-feng Chen, and Chun-fang %J PloS one Gao. "Serum Micrornas as Biomarkers for Hepatocellular Carcinoma in Chinese Patients with Chronic Hepatitis B Virus Infection." 6, no. 12 (2011): e28486.
3. Oksuz, Zehra, Mehmet Sami Serin, Engin Kaplan, Aylin Dogen, Seda Tezcan, Gonul Aslan, Gurol Emekdas, Orhan Sezgin, Engin Altintas, and Eyup Naci Tiftik. "Serum Micrornas; Mir-30c-5p, Mir-223-3p, Mir-302c-3p and Mir-17-5p Could Be Used as Novel Non-Invasive Biomarkers for Hcv-Positive Cirrhosis and Hepatocellular Carcinoma." *Molecular Biology Reports* 42, no. 3 (2015): 713-20.
4. Zheng, Hao, Angela E Zou, Maarouf A Saad, Xiao Qi Wang, James G Kwok, Avinaash Korrapati, Pinxue Li, Tatiana Kisseleva, Jessica Wang-Rodriguez, and Weg M %J PloS one Ongkeko. "Alcohol-Dysregulated Micrornas in Hepatitis B Virus-Related Hepatocellular Carcinoma." 12, no. 5 (2017): e0178547.
5. Li, Lihua, Zijan Guo, Juanhua Wang, Yong Mao, Qi %J Digestive diseases Gao, and sciences. "Serum Mir-18a: A Potential Marker for Hepatitis B Virus-Related Hepatocellular Carcinoma Screening." 57, no. 11 (2012): 2910-16.
6. Zhou, Jian, Lei Yu, Xue Gao, Jie Hu, Jiping Wang, Zhi Dai, Jie-Fei Wang, Zhiyong Zhang, Shaohua Lu, and Xiaowu %J J Clin Oncol Huang. "Plasma Microrna Panel to Diagnose Hepatitis B Virus-Related Hepatocellular Carcinoma." 29, no. 36 (2011): 4781-88.
7. Yu, Guifang, Xuezhu Chen, Shudi Chen, Weipeng Ye, Kailian Hou, and Min Liang. "Mir-19a, Mir-122 and Mir-223 Are Differentially Regulated by Hepatitis B Virus X Protein and Involve in Cell Proliferation in Hepatoma Cells." *Journal of Translational Medicine* 14, no. 1 (2016): 122.
8. Bandopadhyay, Manikankana, Arup Banerjee, Neelakshi Sarkar, Rajesh Panigrahi, Sibnarayan Datta, Ananya Pal, Shivram Prasad Singh, Avik Biswas, Shekhar Chakrabarti, and Runu Chakravarty. "Tumor Suppressor Micro Rna Mir-145 and Onco Micro Rnas Mir-21 and Mir-222 Expressions Are Differentially Modulated by Hepatitis B Virus X Protein in Malignant Hepatocytes." *BMC Cancer* 14, no. 1 (2014): 721.
9. Zhang, Hua, Xuyi Chen, Bo Yang, Minghua Zhang, and Renjie %J Chinese Journal of Clinical Oncology Wang. "Research on the Serum Level of Microrna-224 in Hepatocellular Carcinoma Patients and Its Clinical Diagnostic Significance." (2014): 576-79.
10. Jiang, Runqiu, Lei Deng, Liang Zhao, Xiangcheng Li, Feng Zhang, Yongxiang Xia, Yun Gao, Xuehao Wang, and Beicheng %J Clinical Cancer Research Sun. "Mir-22 Promotes Hbv-Related Hepatocellular Carcinoma Development in Males." 17, no. 17 (2011): 5593-603.
11. Li, Jian, Boxun Jin, Tiezheng Wang, Wenlei Li, Zhenshun Wang, Haitao Zhang, Yunjun Song, and Ning %J Cancer Biomarkers Li. "Serum Microrna Expression Profiling Identifies Serum Biomarkers for Hcv-Related Hepatocellular Carcinoma." 26, no. 4 (2019): 501-12.
12. Chen, Danlei, Yan Yan, Xinyi Wang, Suzhi Li, Yan Liu, Dandan Yu, Yongjing He, Ruiqing Deng, Yakun Liu, Mei Xu, Jia Luo, Hongjun Gao, and Siying Wang. "Chronic Alcohol Exposure Promotes Hcc Stemness and Metastasis through Β-Catenin/Mir-22-3p/Tet2 Axis." *Aging* 13, no. 10 (2021): 14433-55.
13. Wang, Rongchang, Zheng Yu, Fan Chen, Hongxu Xu, Shunli Shen, Wei Chen, Lianzhou Chen, Qiao Su, Longjuan Zhang, Jiong %J Biomedicine Bi, and Pharmacotherapy. "Mir-300 Regulates the Epithelial-Mesenchymal Transition and Invasion of Hepatocellular Carcinoma by Targeting the Fak/Pi3k/Akt Signaling Pathway." 103 (2018): 1632-42.
14. Lv, Guixiang, Mingjuan Wu, Meijie Wang, Xiaochen Jiang, Jingli Du, Kaili Zhang, Dongliang Li, Ning Ma, Yahui Peng, and Lujing %J Liver international Wang. "Mir-320a Regulates High Mobility Group Box 1 Expression and Inhibits Invasion and Metastasis in Hepatocellular Carcinoma." 37, no. 9 (2017): 1354-64.
15. Li, Huifen, Weihua Huang, and Rongcheng %J Diagnostic Pathology Luo. "Retracted Article: The Microrna-325 Inhibits Hepatocellular Carcinoma Progression by Targeting High Mobility Group Box 1." 10, no. 1 (2015): 1-11.

16. Hu, Shiping, Yun Ran, Wenlin Chen, Yuncheng Zhang, and Yongjian %J Oncology reports Xu. "Microrna-326 Inhibits Cell Proliferation and Invasion, Activating Apoptosis in Hepatocellular Carcinoma by Directly Targeting Lim and Sh3 Protein 1." 38, no. 3 (2017): 1569-78.
17. Youssef, S. S., A. Elfiky, M. M. Nabeel, H. I. Shousha, T. Elbaz, D. Omran, M. S. Marie, M. A. Elzahry, A. Abul-Fotouh, A. Hashem, M. F. Guda, and A. O. Abdelaziz. "Assessment of Circulating Levels of Microrna-326, Microrna-424, and Microrna-511 as Biomarkers for Hepatocellular Carcinoma in Egyptians." *World J Hepatol* 14, no. 8 (2022): 1562-75.
18. Meng, Fan-Long, Wei Wang, and Wei-Dong Jia. "Diagnostic and Prognostic Significance of Serum Mir-24-3p in Hbv-Related Hepatocellular Carcinoma." *Medical Oncology* 31, no. 9 (2014): 177.
19. Rashad, Nearmeen M., Amal S. El-Shal, Sally M. Shalaby, and Salem Y. Mohamed. "Serum Mirna-27a and Mirna-18b as Potential Predictive Biomarkers of Hepatitis C Virus-Associated Hepatocellular Carcinoma." *Molecular and Cellular Biochemistry* 447, no. 1 (2018): 125-36.
20. Zhao, X, GQ Chen, and GM %J Eur Rev Med Pharmacol Sci Cao. "Abnormal Expression and Mechanism of Mir-330-3p/Btg1 Axis in Hepatocellular Carcinoma." 23, no. 16 (2019): 6888-98.
21. Shi, Xiaomin, Fei %J Molecular Teng, and Cellular Biochemistry. "Down-Regulated Mir-28-5p in Human Hepatocellular Carcinoma Correlated with Tumor Proliferation and Migration by Targeting Insulin-Like Growth Factor-1 (Igf-1)." 408, no. 1 (2015): 283-93.
22. Yu, M., H. Xue, Y. Wang, Q. Shen, Q. Jiang, X. Zhang, K. Li, M. Jia, J. Jia, J. Xu, and Y. Tian. "Mir-345 Inhibits Tumor Metastasis and Emt by Targeting Irf1-Mediated Mtor/Stat3/Akt Pathway in Hepatocellular Carcinoma." *Int J Oncol* 50, no. 3 (2017): 975-83.
23. Xiao, Zhihua, Yijun Wang, Hao %J Cell Ding, and bioscience. "Xpd Suppresses Cell Proliferation and Migration Via Mir-29a-3p-Mdm2/Pdgf-B Axis in Hcc." 9, no. 1 (2019): 1-12.
24. Cheng, Y, L Qiu, GL He, L Cai, BJ Peng, YL Cao, and MX %J Eur Rev Med Pharmacol Sci Pan. "Microrna-361-5p Suppresses the Tumorigenesis of Hepatocellular Carcinoma through Targeting Wt1 and Suppressing Wnt/Beta-Cadherin Pathway." 23, no. 20 (2019): 8823-32.
25. LiWF, DaiH %J TumourBiol. "Overexpressionofmicrorna◎ 30a◎ 5pinhibitslivercancercellproliferationandinducesapoptosisby Targetingmtdh/Pten/Aktpathway." 37 (2016): 5885.
26. Ni, F., H. Zhao, H. Cui, Z. Wu, L. Chen, Z. Hu, C. Guo, Y. Liu, Z. Chen, X. Wang, D. Chen, H. Wei, and S. Wang. "Microrna-362-5p Promotes Tumor Growth and Metastasis by Targeting Cyld in Hepatocellular Carcinoma." *Cancer Lett* 356, no. 2 Pt B (2015): 809-18.
27. Fu, Xiao, Mengjie Liu, Shengyang Qu, Jiequn Ma, Yamin Zhang, Tingting Shi, Hongqing Wen, Yujuan Yang, Shuhong Wang, Jing %J Journal of Experimental Wang, and Clinical Cancer Research. "Exosomal Microrna-32-5p Induces Multidrug Resistance in Hepatocellular Carcinoma Via the Pi3k/Akt Pathway." 37, no. 1 (2018): 1-18.
28. He, Yongkang, and Xiaofeng %J Yonsei medical journal He. "Microrna-370 Regulates Cellepithelial-Mesenchymal Transition, Migration, Invasion, and Prognosis of Hepatocellular Carcinoma by Targeting Gucd1." 60, no. 3 (2019): 267-76.
29. Li, Na, Hanjiang Fu, Yi Tie, Zheng Hu, Wei Kong, Yongge Wu, and Xiaofei %J Cancer letters Zheng. "Mir-34a Inhibits Migration and Invasion by Down-Regulation of C-Met Expression in Human Hepatocellular Carcinoma Cells." 275, no. 1 (2009): 44-53.
30. Bai, Pei-song, Peng Hou, and Ying Kong. "Hepatitis B Virus Promotes Proliferation and Metastasis in Male Chinese Hepatocellular Carcinoma Patients through the Lef-1/Mir-371a-5p/Srcin1/Pleiotrophin/Slug Pathway." *Experimental Cell Research* 370, no. 1 (2018): 174-88.
31. Wang, Yan, Chun-Mei Wang, Zhen-Zhong Jiang, Xiao-Jian Yu, Chun-Guang Fan, Fei-Fei Xu, Qing Zhang, Li Li, Rui-Feng Li, Wen-Sheng Sun, Zhen-Hai Zhang, and Yu-Gang Liu. "Microrna-34c Targets Tgfb-Induced Factor Homeobox 2, Represses Cell Proliferation and Induces Apoptosis in Hepatitis B Virus-Related Hepatocellular Carcinoma." *Oncol Lett* 10, no. 5 (2015): 3095-102.
32. Li, Hongbin, Nan Wang, Yuntian Xu, Xiao Chang, Jing Ke, and Jun %J Bioengineered Yin. "Upregulating Microrna-373-3p Promotes Apoptosis and Inhibits Metastasis of Hepatocellular Carcinoma Cells." 13, no. 1 (2022): 1304-19.
33. Ohta, Katsuya, Hiromitsu Hoshino, Jinhua Wang, Shigeshi Ono, Yuuki Iida, Keisuke Hata, Sharon K Huang, Steven Colquhoun, and Dave SB %J Oncotarget Hoon. "Microrna-93 Activates C-Met/Pi3k/Akt Pathway Activity in Hepatocellular Carcinoma by Directly Inhibiting Pten and Cdkn1a." 6, no. 5 (2015): 3211.
34. Zhang, Weilu, Ting Fu, Zhenjun Guo, Ye Zhang, Lei Zhang, Haixia Su, Yong Long, Zhaohua Ji, Yongping Yan, and Zhongjun %J BioMed research international Shao. "Serum Mir-375 Levels Are Closely Related to Disease Progression from Hbv Infection to Hbv-Related Hepatocellular Carcinoma." 2020 (2020).

35. Ye, Jian, Yufeng Yao, Qixue Song, Sisi Li, Zhenkun Hu, Yubing Yu, Changqing Hu, Xingwen Da, Hui Li, and Qiuyun %J Scientific reports Chen. "Up-Regulation of Mir-95-3p in Hepatocellular Carcinoma Promotes Tumorigenesis by Targeting P21 Expression." 6, no. 1 (2016): 1-12.
36. Bai, Pei-song, Nan Xia, Hong Sun, Ying %J Journal of Cellular Kong, and Molecular Medicine. "Pleiotrophin, a Target of Mir-384, Promotes Proliferation, Metastasis and Lipogenesis in Hbv-Related Hepatocellular Carcinoma." 21, no. 11 (2017): 3023-43.
37. Chen, Yueming, Xueyan Dong, Daojun Yu, and Xianjun Wang. "Serum Mir-96 Is a Promising Biomarker for Hepatocellular Carcinoma in Patients with Chronic Hepatitis B Virus Infection." *International journal of clinical and experimental medicine* 8, no. 10 (2015): 18462-68.
38. Du, Huimin, Qing Xu, Sheng Xiao, Zhenru Wu, Jianping Gong, Changan Liu, Guosheng Ren, and Hao %J Life sciences Wu. "Microrna-424-5p Acts as a Potential Biomarker and Inhibits Proliferation and Invasion in Hepatocellular Carcinoma by Targeting Trim29." 224 (2019): 1-11.
39. Li, Dong, Xingguang Liu, Li Lin, Jin Hou, Nan Li, Chunmei Wang, Pin Wang, Qian Zhang, Peng Zhang, and Weiping %J Journal of Biological Chemistry Zhou. "Microrna-99a Inhibits Hepatocellular Carcinoma Growth and Correlates with Prognosis of Patients with Hepatocellular Carcinoma." 286, no. 42 (2011): 36677-85.
40. Sun, Kexin, Tiancai Zeng, Dong Huang, Zizhong Liu, Shang Huang, Jiong Liu, and Zhenfan %J FEBS open bio Qu. "Microrna-431 Inhibits Migration and Invasion of Hepatocellular Carcinoma Cells by Targeting the Zeb1-Mediated Epithelial–Mensenchymal Transition." 5 (2015): 900-07.
41. Ambade, Aditya, Abhishek Satishchandran, and Gyongyi Szabo. "Alcoholic Hepatitis Accelerates Early Hepatobiliary Cancer by Increasing Stemness and Mir-122-Mediated Hif-1α Activation." *Scientific Reports* 6, no. 1 (2016): 21340.
42. Tang, Hui, Jianwen Zhang, Zhenyu Yu, Linsen Ye, Kun Li, Fan Ding, Xiao Feng, Wei %J Technology in cancer research Meng, and treatment. "Mir-452-3p: A Potential Tumor Promoter That Targets the Cpeb3/Egfr Axis in Human Hepatocellular Carcinoma." 16, no. 6 (2017): 1136-49.
43. Huang, Xin-Ping, Jin Hou, Xiao-Yun Shen, Chao-Yuan Huang, Xue-Hui Zhang, Yu-An Xie, and Xiao-Ling %J The FEBS journal Luo. "Micro Rna-486-5p, Which Is Downregulated in Hepatocellular Carcinoma, Suppresses Tumor Growth by Targeting Pik3r1." 282, no. 3 (2015): 579-94.
44. El-Ahwany, Eman GE, Lobna Mourad, Mona MK Zoheiry, Hoda Abu-Taleb, Marwa Hassan, Raafat Atta, Moataz Hassanien, and Suher %J Archives of Medical Science: AMS Zada. "Microrna-122a as a Non-Invasive Biomarker for Hcv Genotype 4-Related Hepatocellular Carcinoma in Egyptian Patients." 15, no. 6 (2019): 1454.
45. Xu, Bin, Tangpeng Xu, Huali Liu, Qian Min, Shidong Wang, and Qibin %J Pharmacology Song. "Mir-490-5p Suppresses Cell Proliferation and Invasion by Targeting Bub1 in Hepatocellular Carcinoma Cells." 100, no. 5-6 (2017): 269-82.
46. Felgendreff, Philipp, Nathanael Raschzok, Kerstin Kunze, Annekatrin Leder, Steffen Lippert, Sergej Klunk, Hans-Michael Tautenhahn, Hans-Michael Hau, Rosa Bianca Schmuck, and Anja Reutzel-Selke. "Tissue-Based Mirna Mapping in Alcoholic Liver Cirrhosis: Different Profiles in Cirrhosis with or without Hepatocellular Carcinoma." *Biomarkers* 25, no. 1 (2020): 62-68.
47. Zhou, Yun, Yuan Li, Jing Ye, Rongrong Jiang, Han Yan, Xiaojun Yang, Qizhan Liu, and Jianping %J Liver international Zhang. "Micro Rna-491 Is Involved in Metastasis of Hepatocellular Carcinoma by Inhibitions of Matrix Metalloproteinase and Epithelial to Mesenchymal Transition." 33, no. 8 (2013): 1271-80.
48. Liu, Zhikui, Changwei Dou, Bowen Yao, Meng Xu, Linglong Ding, Yufeng Wang, Yuli Jia, Qing Li, Hongyong Zhang, and Kangsheng %J Oncotarget Tu. "Methylation-Mediated Repression of Microrna-129-2 Suppresses Cell Aggressiveness by Inhibiting High Mobility Group Box 1 in Human Hepatocellular Carcinoma." 7, no. 24 (2016): 36909.
49. Ding, Wei, Hongbo Tan, Xuemei Li, Yue Zhang, Fang Fang, Yuanyuan Tian, Jin Li, and Xinghua %J Cancer biomarkers Pan. "Microrna-493 Suppresses Cell Proliferation and Invasion by Targeting Zfx in Human Hepatocellular Carcinoma." 22, no. 3 (2018): 427-34.
50. Morishita, Asahiro, Koji Fujita, Hisakazu Iwama, Taiga Chiyo, Shintaro Fujihara, Kyoko Oura, Tomoko Tadokoro, Shima Mimura, Takako Nomura, Joji %J American Journal of Physiology-Gastrointestinal Tani, and Liver Physiology. "Role of Microrna-210-3p in Hepatitis B Virus-Related Hepatocellular Carcinoma." 318, no. 3 (2020): G401-G09.
51. Li, Hui, Zhigang Xiang, Yan Liu, Bin Xu, and Jianzhou %J Oncology research Tang. "Microrna-133b Inhibits Proliferation, Cellular Migration, and Invasion Via Targeting Lasp1 in Hepatocarcinoma Cells." 25, no. 8 (2017): 1269.
52. Xiao, Yao, Qinggang Tian, Jiantai He, Ming Huang, Chao Yang, Liansheng %J OncoTargets Gong, and therapy. "Mir-503 Inhibits Hepatocellular Carcinoma Cell Growth Via Inhibition of Insulin-Like Growth Factor 1 Receptor." 9 (2016): 3535.

53. Wang, Jiachen, Zhao Wang, Jiaxiang Yuan, Qun Wang, Xinsheng %J OncoTargets Shen, and therapy. "Upregulation of Mir-137 Expression Suppresses Tumor Growth and Progression Via Interacting with Dnmt3a through Inhibiting the Pten/Akt Signaling in Hcc." 14 (2021): 165.
54. Hu, Xiu-Mei, Xiao-Hui Yan, Yan-Wei Hu, Jin-Lan Huang, Shun-Wang Cao, Ting-Yu Ren, Yue-Ting Tang, Li Lin, Lei Zheng, and Qian %J Hepatology Research Wang. "Mirna-548p Suppresses Hepatitis B Virus X Protein Associated Hepatocellular Carcinoma by Downregulating Oncoprotein Hepatitis B X-Interacting Protein." 46, no. 8 (2016): 804-15.
55. Lin, Y. X., X. B. Wu, C. W. Zheng, Q. L. Zhang, G. Q. Zhang, K. Chen, Q. Zhan, and F. M. An. "Mechanistic Investigation on the Regulation of Fabp1 by the Il-6/Mir-603 Signaling in the Pathogenesis of Hepatocellular Carcinoma." *Biomed Res Int* 2021 (2021): 8579658.
56. Song, Wei-Hua, Xiao-Jun Feng, Shao-Juan Gong, Jian-Ming Chen, Shou-Mei Wang, Dong-Juan Xing, Ming-Hua Zhu, Shu-Hui Zhang, Ai-Min %J Cancer biology Xu, and therapy. "Microrna-622 Acts as a Tumor Suppressor in Hepatocellular Carcinoma." 16, no. 12 (2015): 1754-63.
57. Luo, G., Y. L. Chao, B. Tang, B. S. Li, Y. F. Xiao, R. Xie, S. M. Wang, Y. Y. Wu, H. Dong, X. D. Liu, and S. M. Yang. "Mir-149 Represses Metastasis of Hepatocellular Carcinoma by Targeting Actin-Regulatory Proteins Ppm1f." *Oncotarget* 6, no. 35 (2015): 37808-23.
58. Huang, Weifeng, Qingsong Chen, Jiangweng Dai, Yuke Zhang, Yan Yi, Xufu Wei, and Zhongjun %J Journal of Gastrointestinal Oncology Wu. "Mir-744-5p Suppresses Tumor Proliferation and Metastasis by Targeting Transforming Growth Factor-Beta 1 (Tgf-Β1) in Hepatocellular Carcinoma (Hcc)." 12, no. 4 (2021): 1811.
59. Li, Tao, Junjie Xie, Chuan Shen, Dongfeng Cheng, Yuan Shi, Zhichong Wu, Qian Zhan, Xiaxing Deng, Hao Chen, and Baiyong %J PloS one Shen. "Mir-150-5p Inhibits Hepatoma Cell Migration and Invasion by Targeting Mmp14." 9, no. 12 (2014): e115577.
60. Miquelestorena-Standley, Elodie, Anne Tallet, Christine Collin, Eric Piver, Anne De Muret, Ephrem Salamé, Pascal Bourlier, Thibault Kervarrec, Serge Guyétant, and Jean-Christophe %J Hepatology Research Pagès. "Interest of Variations in Microrna-152 and-122 in a Series of Hepatocellular Carcinomas Related to Hepatitis C Virus Infection." 48, no. 7 (2018): 566-73.
61. Yan, TH, Chunguang Qiu, Jifan Sun, and WH %J Eur Rev Med Pharmacol Sci Li. "Mir-877-5p Suppresses Cell Growth, Migration and Invasion by Targeting Cyclin Dependent Kinase 14 and Predicts Prognosis in Hepatocellular Carcinoma." 22, no. 10 (2018): 3038-46.
62. Zhang, Yiliang, Wei Wei, Na Cheng, Kaihui Wang, Bin Li, Xiaoqing Jiang, and Shuhan %J Hepatology Sun. "Hepatitis C Virus-Induced up-Regulation of Microrna-155 Promotes Hepatocarcinogenesis by Activating Wnt Signaling." 56, no. 5 (2012): 1631-40.
63. Liu, Zhikui, Yufeng Wang, Changwei Dou, Liankang Sun, Qing Li, Liang Wang, Qiuran Xu, Wei Yang, Qingguang Liu, Kangsheng %J Journal of Experimental Tu, and Clinical Cancer Research. "Microrna-1468 Promotes Tumor Progression by Activating Ppar-Γ-Mediated Akt Signaling in Human Hepatocellular Carcinoma." 37, no. 1 (2018): 1-14.
64. Shaheen, Noha Mohamed Hosni, Naglaa Zayed, Nermine Magdi Riad, Hend H. Tamim, Rasha Mohamed Hosny Shahin, Dalia A. Labib, Suzan Mahrous Elsheikh, Reham Abdel Moneim, Ayman Yosry, and Rania H. khalifa. "Role of Circulating Mir-182 and Mir-150 as Biomarkers for Cirrhosis and Hepatocellular Carcinoma Post Hcv Infection in Egyptian Patients." *Virus Research* 255 (2018): 77-84.
65. Ding, Dong, Yaodong Zhang, Renjie Yang, Xing Wang, Guwei Ji, Liqun Huo, Zicheng Shao, and Xiangcheng %J BioMed Research International Li. "Mir-940 Suppresses Tumor Cell Invasion and Migration Via Regulation of Cxcr2 in Hepatocellular Carcinoma." 2016 (2016).
66. Dou, C., Z. Liu, M. Xu, Y. Jia, Y. Wang, Q. Li, W. Yang, X. Zheng, K. Tu, and Q. Liu. "Mir-187-3p Inhibits the Metastasis and Epithelial-Mesenchymal Transition of Hepatocellular Carcinoma by Targeting S100a4." *Cancer Lett* 381, no. 2 (2016): 380-90.
67. Zhang, Chao, Peng Liu, and Chuanbao %J Journal of Cellular Biochemistry Zhang. "Hepatitis B Virus X Protein Upregulates Alpha-Fetoprotein to Promote Hepatocellular Carcinoma by Targeting Mir-1236 and Mir-329." 121, no. 3 (2020): 2489-99.
68. Ghosh, Amit, Alip Ghosh, Somenath Datta, Debanjali Dasgupta, Soumyajit Das, Sukanta Ray, Subash Gupta, Simanti Datta, Abhijit Chowdhury, and Raghunath %J International journal of cancer Chatterjee. "Hepatic Mi R-126 Is a Potential Plasma Biomarker for Detection of Hepatitis B Virus Infected Hepatocellular Carcinoma." 138, no. 11 (2016): 2732-44.
69. Lu, Junhao, Licheng Tang, Yuqiang Xu, Kuikui Ge, Jinjiang Huang, Meigang Gu, Jiang Zhong, and Qingshan %J Journal of Cellular Biochemistry Huang. "Mir-1287 Suppresses the Proliferation, Invasion, and Migration in Hepatocellular Carcinoma by Targeting Pik3r3." 119, no. 11 (2018): 9229-38.
70. Xu, Qiuran, Xin Liu, Zhikui Liu, Zhenyu Zhou, Yufeng Wang, Jianfeng Tu, Lijie Li, Hangxing Bao, Liu Yang, and Kangsheng %J Molecular cancer Tu. "Microrna-1296 Inhibits Metastasis and Epithelial-

Mesenchymal Transition of Hepatocellular Carcinoma by Targeting Srpk1-Mediated Pi3k/Akt Pathway." 16, no. 1 (2017): 1-15.

71. Motawi, Tarek K, Olfat G Shaker, Shohda A El-Maraghy, and Mahmoud A %J PloS one Senousy. "Serum Micrornas as Potential Biomarkers for Early Diagnosis of Hepatitis C Virus-Related Hepatocellular Carcinoma in Egyptian Patients." 10, no. 9 (2015): e0137706.
72. Felgendreff, Philipp, Nathanael Raschzok, Kerstin Kunze, Annekatrin Leder, Steffen Lippert, Sergej Klunk, Hans-Michael Tautenhahn, Hans-Michael Hau, Rosa Bianca Schmuck, and Anja %J Biomarkers Reutzel-Selke. "Tissue-Based Mirna Mapping in Alcoholic Liver Cirrhosis: Different Profiles in Cirrhosis with or without Hepatocellular Carcinoma." 25, no. 1 (2020): 62-68.
73. Yao, Bowen, Yazhao Li, Liang Wang, Tianxiang Chen, Yongshen Niu, Qingguang Liu, Zhikui %J Artificial cells Liu, nanomedicine,, and biotechnology. "Microrna-3194-3p Inhibits Metastasis and Epithelial-Mesenchymal Transition of Hepatocellular Carcinoma by Decreasing Wnt/B-Catenin Signaling through Targeting Bcl9." 47, no. 1 (2019): 3885-95.
74. Zhang, Tao, Junping Zhang, Ming Cui, Fabao Liu, Xiaona You, Yumei Du, Yuen Gao, Shuai Zhang, Zhanping Lu, and Lihong %J Neoplasia Ye. "Hepatitis B Virus X Protein Inhibits Tumor Suppressor Mir-205 through Inducing Hypermethylation of Mir-205 Promoter to Enhance Carcinogenesis." 15, no. 11 (2013): 1282-IN26.
75. Zhao, Xinyang, Qilong Song, Ge Miao, Xinfeng %J OncoTargets Zhu, and therapy. "Microrna-3651 Promotes the Growth and Invasion of Hepatocellular Carcinoma Cells by Targeting Pten." 12 (2019): 7045.
76. Han, Shaoshan, Yu Shi, Liankang Sun, Zhikui Liu, Tao Song, and Qingguang %J International journal of biological sciences Liu. "Mir-4319 Induced an Inhibition of Epithelial-Mesenchymal Transition and Prevented Cancer Stemness of Hcc through Targeting Foxq1." 15, no. 13 (2019): 2936.
77. Deng, Biao, Lei Qu, Jinfang Li, Jiaqing Fang, Shouwen Yang, Zhongwei Cao, Zhechuan Mei, and Xing %J Scientific reports Sun. "Mirna-211 Suppresses Cell Proliferation, Migration and Invasion by Targeting Sparc in Human Hepatocellular Carcinoma." 6, no. 1 (2016): 1-12.
78. Xie, Yingjun, Jian Du, Zefeng Liu, Dan Zhang, Xiaoxiao Yao, Yongsheng %J Journal of Experimental Yang, and Clinical Cancer Research. "Mir-6875-3p Promotes the Proliferation, Invasion and Metastasis of Hepatocellular Carcinoma Via Btg2/Fak/Akt Pathway." 38, no. 1 (2019): 1-14.
79. Ruivo, Carolina F, Bárbara Adem, Miguel Silva, and Sónia A %J Cancer research Melo. "The Biology of Cancer Exosomes: Insights and New Perspectivesbiology of Cancer Exosomes." 77, no. 23 (2017): 6480-88.
80. Jun, Li, Gu Yang, Liu %J Biomedicine Zhisu, and Pharmacotherapy. "The Utility of Serum Exosomal Micrornas in Hepatocellular Carcinoma." 111 (2019): 1221-27.
81. Barile, Lucio, Tiziano Moccetti, Eduardo Marbán, and Giuseppe %J European Heart Journal Vassalli. "Roles of Exosomes in Cardioprotection." 38, no. 18 (2017): 1372-79.
82. Pan, Jing-Hua, Hong Zhou, Xiao-Xu Zhao, Hui Ding, Wei Li, Li Qin, and Yun-Long %J International journal of molecular medicine Pan. "Role of Exosomes and Exosomal Micrornas in Hepatocellular Carcinoma: Potential in Diagnosis and Antitumour Treatments." 41, no. 4 (2018): 1809-16.
83. Li, Shuangshuang, Jiping Yao, Mingjie Xie, Yanning Liu, Min %J Journal of Hematology Zheng, and Oncology. "Exosomal Mirnas in Hepatocellular Carcinoma Development and Clinical Responses." 11, no. 1 (2018): 1-9.
84. Liu, Chenbin, Han Wu, Yinqi Mao, Wei Chen, and Shuying %J Cancer Cell International Chen. "Exosomal Micrornas in Hepatocellular Carcinoma." 21, no. 1 (2021): 1-12.
85. Liu, Wei-hui, Li-na Ren, Xing Wang, Tao Wang, Ning Zhang, Yuan Gao, Hao Luo, Nalu Navarro-Alvarez, Li-jun %J Journal of cancer research Tang, and clinical oncology. "Combination of Exosomes and Circulating Micrornas May Serve as a Promising Tumor Marker Complementary to Alpha-Fetoprotein for Early-Stage Hepatocellular Carcinoma Diagnosis in Rats." 141, no. 10 (2015): 1767-78.
86. Sohn, Won, Jonghwa Kim, So Hee Kang, Se Ra Yang, Ju-Yeon Cho, Hyun Chin Cho, Sang Goon Shim, Yong-Han %J Experimental Paik, and molecular medicine. "Serum Exosomal Micrornas as Novel Biomarkers for Hepatocellular Carcinoma." 47, no. 9 (2015): e184-e84.
87. Liu, Wanbo, Sheng Chen, and Bing %J Pediatric surgery international Liu. "Diagnostic and Prognostic Values of Serum Exosomal Microrna-21 in Children with Hepatoblastoma: A Chinese Population-Based Study." 32, no. 11 (2016): 1059-65.
88. Jiao, Chenwei, Xiaohu Jiao, Anzhi Zhu, Juntao Ge, and Xiaoqing %J Journal of pediatric surgery Xu. "Exosomal Mir-34s Panel as Potential Novel Diagnostic and Prognostic Biomarker in Patients with Hepatoblastoma." 52, no. 4 (2017): 618-24.
89. Xue, Xiaofeng, Xiaona Wang, Yubin Zhao, Rongkuan Hu, Lei %J Biochemical Qin, and biophysical research communications. "Exosomal Mir-93 Promotes Proliferation and Invasion in Hepatocellular Carcinoma by Directly Inhibiting Timp2/Tp53inp1/Cdkn1a." 502, no. 4 (2018): 515-21.

90. Fan, Baochang, FX Reymond Sutandy, Guan-Da Syu, Stefani Middleton, Guanghui Yi, Kuan-Yi Lu, Chien-Sheng Chen, C Cheng %J Molecular Kao, and Cellular Proteomics. "Heterogeneous Ribonucleoprotein K (Hnrnp K) Binds Mir-122, a Mature Liver-Specific Microrna Required for Hepatitis C Virus Replication." 14, no. 11 (2015): 2878-86.
91. Aucher, Anne, Dominika Rudnicka, and Daniel M %J The Journal of Immunology Davis. "Micrornas Transfer from Human Macrophages to Hepato-Carcinoma Cells and Inhibit Proliferation." 191, no. 12 (2013): 6250-60.
92. Xu, Guili, Peng Zhang, Hansi Liang, Yunhua Xu, Jian Shen, Wansheng Wang, Mingming Li, Jintao Huang, Caifang Ni, and Xueguang Zhang. "Circular Rna Hsa_Circ_0003288 Induces Emt and Invasion by Regulating Hsa_Circ_0003288/Mir-145/Pd-L1 Axis in Hepatocellular Carcinoma." *Cancer cell international* 21, no. 1 (2021): 1-13.
93. Singh, Anurag, and JEMT Settleman. "Emt, Cancer Stem Cells and Drug Resistance: An Emerging Axis of Evil in the War on Cancer." *Oncogene* 29, no. 34 (2010): 4741-51.
94. Chen, Yan, Shuhua Li, Yinbin Wei, Zhihong Xu, and Xiongfei Wu. "Circ-Rnf13, as an Oncogene, Regulates Malignant Progression of Hbv-Associated Hepatocellular Carcinoma Cells and Hbv Infection through Cerna Pathway of Circ-Rnf13/Mir-424-5p/Tgif2." *Bosnian Journal of Basic Medical Sciences* 21, no. 5 (2021): 555.
95. Anderson, Anoush E, Kenichiro Taniguchi, Yi Hao, Tiffany A Melhuish, Anant Shah, Stephen D Turner, Ann E Sutherland, and David Wotton. "Tgif1 and Tgif2 Repress Expression of the Rabgap Evi5l." *Molecular and cellular biology* 37, no. 5 (2017): e00527-16.
96. Cao, Shuang, Guohua Wang, Jia Wang, Cheng Li, and Le Zhang. "Hsa_Circ_101280 Promotes Hepatocellular Carcinoma by Regulating Mir-375/Jak2." *Immunology and Cell Biology* 97, no. 2 (2019): 218-28.
97. Sonohara, Fuminori, Shuji Nomoto, Yoshikuni Inokawa, Mitsuhiro Hishida, Nao Takano, Mitsuro Kanda, Yoko Nishikawa, Tsutomu Fujii, Masahiko Koike, and Hiroyuki Sugimoto. "High Expression of Janus Kinase 2 in Background Normal Liver Tissue of Resected Hepatocellular Carcinoma Is Associated with Worse Prognosis." *Oncology reports* 33, no. 2 (2015): 767-73.
98. Li, Liheng, Chengjiang Xiao, Ke He, and Guoan Xiang. "Circ_0072088 Promotes Progression of Hepatocellular Carcinoma by Activating Jak2/Stat3 Signaling Pathway Via Mir-375." *IUBMB life* 73, no. 9 (2021): 1153-65.
99. Chen, Wei, Yingyao Quan, Shaoyi Fan, Hua Wang, Jinyu Liang, Li Huang, Liuhua Chen, Qi Liu, Peiheng He, and Yibiao %J Cancer letters Ye. "Exosome-Transmitted Circular Rna Hsa_Circ_0051443 Suppresses Hepatocellular Carcinoma Progression." 475 (2020): 119-28.
100. Wang, Yi-Dong, N Cai, XL Wu, HZ Cao, LL Xie, and PS Zheng. "Oct4 Promotes Tumorigenesis and Inhibits Apoptosis of Cervical Cancer Cells by Mir-125b/Bak1 Pathway." *Cell death & disease* 4, no. 8 (2013): e760-e60.
101. Su, Xiaotong, Jutong Su, Hua He, Yong Zhan, Haichao %J Biomedicine Liu, and Pharmacotherapy. "Hsa_Circ_0070269 Inhibits Hepatocellular Carcinoma Progression through Modulating Mir-182/Nptx1 Axis." 120 (2019): 109497.
102. Su, Xiaotong, Jutong Su, Hua He, Yong Zhan, and Haichao Liu. "Hsa_Circ_0070269 Inhibits Hepatocellular Carcinoma Progression through Modulating Mir-182/Nptx1 Axis." *Biomedicine & Pharmacotherapy* 120 (2019): 109497.
103. Boles, Nathan C, Sarah E Hirsch, Sheila Le, Barbara Corneo, Fadi Najm, Andrew P Minotti, Qingjie Wang, Steven Lotz, Paul J Tesar, and Christopher A Fasano. "Nptx1 Regulates Neural Lineage Specification from Human Pluripotent Stem Cells." *Cell reports* 6, no. 4 (2014): 724-36.
104. Cao, Man-Qing, A-Bin You, Xiao-Dong Zhu, Wei Zhang, Yuan-Yuan Zhang, Shi-Zhe Zhang, Ke-wei Zhang, Hao Cai, Wen-Kai Shi, and Xiao-Long Li. "Mir-182-5p Promotes Hepatocellular Carcinoma Progression by Repressing Foxo3a." *Journal of hematology & oncology* 11, no. 1 (2018): 1-12.
105. Huang, Xiu-Yan, Zi-Li Huang, Bin Xu, Zi Chen, Thomas Joseph Re, Qi Zheng, Zhao-You Tang, and Xin-Yu Huang. "Elevated Mtss1 Expression Associated with Metastasis and Poor Prognosis of Residual Hepatitis B-Related Hepatocellular Carcinoma." *Journal of Experimental & Clinical Cancer Research* 35, no. 1 (2016): 1-13.
106. Li, Rong, Yinan Deng, Jinliang Liang, Zhongying Hu, Xuejiao Li, Huanyi Liu, Guoying Wang, Binsheng Fu, Tong Zhang, and Qi %J Cellular Oncology Zhang. "Circular Rna Circ-102,166 Acts as a Sponge of Mir-182 and Mir-184 to Suppress Hepatocellular Carcinoma Proliferation and Invasion." 44, no. 2 (2021): 279-95.
107. Wu, Minhua, Xubin Deng, Yu Zhong, Li Hu, Xiujuan Zhang, Yanqin Liang, Xiaofang Li, and Xiaoxia %J Oncology Research Ye. "Maff Is Regulated Via the Circ-Itch/Mir-224-5p Axis and Acts as a Tumor Suppressor in Hepatocellular Carcinoma." 28, no. 3 (2020): 299.

108. Mao, Chuangjie, Huitao Wen, Yiqian Zhang, Ge Yu, and Qui Ge. "Cirs-7 Enhances the Progression of Hepatocellular Carcinoma through Mir-944/Nox4 Pathway." *Critical Reviews™ in Eukaryotic Gene Expression* 32, no. 7 (2022).
109. Kim, Young-Mee, Seok-Jo Kim, Ryosuke Tatsunami, Hisao Yamamura, Tohru Fukai, and Masuko Ushio-Fukai. "Ros-Induced Ros Release Orchestrated by Nox4, Nox2, and Mitochondria in Vegf Signaling and Angiogenesis." *American Journal of Physiology-Cell Physiology* 312, no. 6 (2017): C749-C64.
110. Newton, Fay, and Roly %J Genes Megaw. "Mechanisms of Photoreceptor Death in Retinitis Pigmentosa." 11, no. 10 (2020): 1120.
111. Huang, Wei, Feizhou Huang, Chao %J OncoTargets Feng, and therapy. "Circfoxo3 Promotes Adriamycin Resistance through Regulation of Mir-199a-5p/Atp Binding Cassette Subfamily C Member 1 Axis in Hepatocellular Carcinoma." 13 (2020): 5113.
112. Yao, Juntao, Xuan Yao, Tao Tian, Xiao Fu, Wenjuan Wang, Suoni Li, Tingting Shi, Aili Suo, Zhiping Ruan, and Hui Guo. "Abcb5–Zeb1 Axis Promotes Invasion and Metastasis in Breast Cancer Cells." *Oncology Research* 25, no. 3 (2017): 305.
113. Guo, Jianbo, HE Duan, Yan Li, Liang Yang, and LU Yuan. "A Novel Circular Rna Circ-Znf652 Promotes Hepatocellular Carcinoma Metastasis through Inducing Snail-Mediated Epithelial-Mesenchymal Transition by Sponging Mir-203/Mir-502-5p." *Biochemical and biophysical research communications* 513, no. 4 (2019): 812-19.
114. Li, Yuhui, Hongliang Zang, Xue Zhang, Guomin %J Cancer Management Huang, and Research. "Exosomal Circ-Znf652 Promotes Cell Proliferation, Migration, Invasion and Glycolysis in Hepatocellular Carcinoma Via Mir-29a-3p/Gucd1 Axis." 12 (2020): 7739.
115. Bellet, Marina Maria, Danilo Piobbico, Daniela Bartoli, Marilena Castelli, Stefania Pieroni, Cinzia Brunacci, Martina Chiacchiaretta, Rachele Del Sordo, Francesca Fallarino, and Angelo Sidoni. "Nedd4 Controls the Expression of Gucd1, a Protein Upregulated in Proliferating Liver Cells." *Cell Cycle* 13, no. 12 (2014): 1902-11.
116. Wang, Panpan, Yunhuan Zhang, Lugang Deng, Zhi Qu, Peisen Guo, Limin Liu, Zengli Yu, Peixi Wang, and Nan Liu. "The Function and Regulation Network Mechanism of Circrna in Liver Diseases." *Cancer cell international* 22, no. 1 (2022): 1-18.
117. Wei, Hailiang, Shuguang Yan, Yi Hui, Yonggang Liu, Hui Guo, Qian Li, Jingtao Li, and Zhanjie Chang. "Circfat1 Promotes Hepatocellular Carcinoma Progression Via Mir-30a-5p/Reep3 Pathway." *Journal of Cellular and Molecular Medicine* 24, no. 24 (2020): 14561-70.
118. Ambros, Victor, Bonnie Bartel, David P Bartel, Christopher B Burge, James C Carrington, Xuemei Chen, Gideon Dreyfuss, Sean R Eddy, SAM Griffiths-Jones, and Mhairi Marshall. "A Uniform System for Microrna Annotation." *Rna* 9, no. 3 (2003): 277-79.
119. Huang, Guanqun, Min Liang, Haiyan Liu, Jianhong Huang, Peiqing Li, Chong Wang, Yidan Zhang, Ye Lin, and Xianhan Jiang. "Circrna Hsa_Circrna_104348 Promotes Hepatocellular Carcinoma Progression through Modulating Mir-187-3p/Rtkn2 Axis and Activating Wnt/Β-Catenin Pathway." *Cell death & disease* 11, no. 12 (2020): 1-14.
120. Dat, Le Tan, Taisuke Matsuo, Tetsuro Yoshimaru, Soji Kakiuchi, Hisatsugu Goto, Masaki Hanibuchi, Takuya Kuramoto, Yasuhiko Nishioka, Saburo Sone, and Toyomasa Katagiri. "Identification of Genes Potentially Involved in Bone Metastasis by Genome-Wide Gene Expression Profile Analysis of Non-Small Cell Lung Cancer in Mice." *International journal of oncology* 40, no. 5 (2012): 1455-69.
121. He, Shuwei, Zhengwu Guo, Qian Kang, Xu Wang, and Xingmin Han. "Circular Rna Hsa_Circ_0000517 Modulates Hepatocellular Carcinoma Advancement Via the Mir-326/Smad6 Axis." *Cancer cell international* 20, no. 1 (2020): 1-13.
122. Xu, Jian, and Rik Derynck. "Does Smad6 Methylation Control Bmp Signaling in Cancer?" *Cell Cycle* 13, no. 8 (2014): 1209-10.
123. Wang, Xicheng, Xining Wang, Wenxin Li, Qi Zhang, Jie Chen, and Tao Chen. "Up-Regulation of Hsa_Circ_0000517 Predicts Adverse Prognosis of Hepatocellular Carcinoma." *Frontiers in oncology* 9 (2019): 1105.
124. Gong, Jianzhuang, Chenxu Du, Nai Sun, Xingguo Xiao, and Huili Wu. "Circular Rna Hsa_Circ_0005397 Promotes Hepatocellular Carcinoma Progression by Regulating the Mir-326/Pdk2 Axis." *The Journal of Gene Medicine* 23, no. 6 (2021): e3332.
125. Hu, Zhi-Qiang, Shao-Lai Zhou, Jia Li, Zheng-Jun Zhou, Peng-Cheng Wang, Hao-Yang Xin, Li Mao, Chu-Bin Luo, Song-Yang Yu, and Xiao-Wu Huang. "Circular Rna Sequencing Identifies Circasap1 as a Key Regulator in Hepatocellular Carcinoma Metastasis." *Hepatology* 72, no. 3 (2020): 906-22.
126. Tay, Yvonne, John Rinn, and Pier Paolo Pandolfi. "The Multilayered Complexity of Cerna Crosstalk and Competition." *Nature* 505, no. 7483 (2014): 344-52.

127. Wang, Cun, Haojie Jin, Dongmei Gao, Cor Lieftink, Bastiaan Evers, Guangzhi Jin, Zheng Xue, Liqin Wang, Roderick L Beijersbergen, and Wenxin Qin. "Phospho-Erk Is a Biomarker of Response to a Synthetic Lethal Drug Combination of Sorafenib and Mek Inhibition in Liver Cancer." *Journal of hepatology* 69, no. 5 (2018): 1057-65.
128. Yang, Guangsheng, Xin Wang, Bingqi Liu, Zhihua Lu, Zongzhen Xu, Peng Xiu, Zhiqian Liu, and Jie Li. "Circ-Birc6, a Circular Rna, Promotes Hepatocellular Carcinoma Progression by Targeting the Mir-3918/Bcl2 Axis." *Cell Cycle* 18, no. 9 (2019): 976-89.
129. Jia, Benli, Xiaoqiang Yin, Yong Wang, Jin Qian, Yan He, Chuang Yang, Gang Yu, Bing Guo, and Xiangling Meng. "Circrna-Ptn Sponges Mir-326 to Promote Proliferation in Hepatocellular Carcinoma." *OncoTargets and therapy* 13 (2020): 4893.
130. Berasain, Carmen, Josefa Castillo, Jesús Prieto, and Matías A Avila. "New Molecular Targets for Hepatocellular Carcinoma: The Erbb1 Signaling System." *Liver International* 27, no. 2 (2007): 174-85.
131. He, Xin, Zhenqi Zhu, Carla Johnson, John Stoops, Amanda E Eaker, William Bowen, and Marie C DeFrances. "Pik3ip1, a Negative Regulator of Pi3k, Suppresses the Development of Hepatocellular Carcinoma." *Cancer research* 68, no. 14 (2008): 5591-98.
132. Huang, Yu, Wenhao Ge, Yuan Ding, Lufei Zhang, Jiarong Zhou, Yang Kong, Bijun Cui, Bingqiang Gao, Xiaohui Qian, and Weilin %J Cancer cell international Wang. "The Circular Rna Circslc7a11 Functions as a Mir-330-3p Sponge to Accelerate Hepatocellular Carcinoma Progression by Regulating Cyclin-Dependent Kinase 1 Expression." 21, no. 1 (2021): 1-22.
133. Asghar, Uzma, Agnieszka K Witkiewicz, Nicholas C Turner, and Erik S Knudsen. "The History and Future of Targeting Cyclin-Dependent Kinases in Cancer Therapy." *Nature reviews Drug discovery* 14, no. 2 (2015): 130-46.
134. Zhang, Juan, Yuxia Chang, Lijun Xu, and Lijie Qin. "Elevated Expression of Circular Rna Circ_0008450 Predicts Dismal Prognosis in Hepatocellular Carcinoma and Regulates Cell Proliferation, Apoptosis, and Invasion Via Sponging Mir-548p." *Journal Of Cellular Biochemistry* 120, no. 6 (2019): 9487-94.
135. Du, Qiajun, Jie Han, Shan Gao, Shangdi Zhang, and Yunyan Pan. "Hypoxia-Induced Circular Rna Hsa_Circ_0008450 Accelerates Hepatocellular Cancer Progression Via the Mir-431/Akap1 Axis." *Oncology letters* 20, no. 6 (2020): 1-1.
136. Rinaldi, Laura, Maria Sepe, Rossella Delle Donne, Kristel Conte, Antonietta Arcella, Domenica Borzacchiello, Stefano Amente, Fernanda De Vita, Monia Porpora, and Corrado Garbi. "Mitochondrial Akap1 Supports Mtor Pathway and Tumor Growth." *Cell death & disease* 8, no. 6 (2017): e2842-e42.
137. Gao, Jun, Chao Dai, Xin Yu, Xiang-Bao Yin, and Fan %J Molecular Oncology Zhou. "Circ-Tcf4. 85 Silencing Inhibits Cancer Progression through Microrna-486-5p-Targeted Inhibition of Abcf2 in Hepatocellular Carcinoma." 14, no. 2 (2020): 447-61.
138. Liu, Shumin, Shun Zhou, Ling Tian, Enen Guo, Yunxia Luan, Jianzhen Zhang, and Sheng Li. "Genome-Wide Identification and Characterization of Atp-Binding Cassette Transporters in the Silkworm, Bombyx Mori." *BMC genomics* 12, no. 1 (2011): 1-15.
139. Degot, Sebastien, Catherine H Regnier, Corinne Wendling, Marie-Pierre Chenard, Marie-Christine Rio, and Catherine Tomasetto. "Metastatic Lymph Node 51, a Novel Nucleo-Cytoplasmic Protein Overexpressed in Breast Cancer." *Oncogene* 21, no. 28 (2002): 4422-34.
140. Liu, Wei, Changbin Yin, and Yue Liu. "Circular Rna Circ_0091579 Promotes Hepatocellular Carcinoma Proliferation, Migration, Invasion, and Glycolysis through Mir-490-5p/Casc3 Axis." *Cancer Biotherapy & Radiopharmaceuticals* 36, no. 10 (2021): 863-78.
141. Cai, Huajie, Bingren Hu, Ling Ji, Xiaojiao Ruan, and Zhihai Zheng. "Hsa_Circ_0103809 Promotes Cell Proliferation and Inhibits Apoptosis in Hepatocellular Carcinoma by Targeting Mir-490-5p/Sox2 Signaling Pathway." *American journal of translational research* 10, no. 6 (2018): 1690.
142. Lan, Gongbin, Luoyan Yang, Xubiao Xie, Longkai Peng, and Yi Wang. "Microrna-490-5p Is a Novel Tumor Suppressor Targeting C-Fos in Human Bladder Cancer." *Archives of medical science* 11, no. 3 (2015): 561-69.
143. Zhong, Feng, Xinsheng Cheng, Shibo Sun, and Jie Zhou. "Transcriptional Activation of Pd-L1 by Sox2 Contributes to the Proliferation of Hepatocellular Carcinoma Cells." *Oncology reports* 37, no. 5 (2017): 3061-67.
144. Zou, Haibo, Xiangang Xu, Lanyun Luo, Yu Zhang, Le Luo, Yutong Yao, Guangming Xiang, Xiaolun Huang, and Guan Wang. "Retracted Article: Hsa_Circ_0101432 Promotes the Development of Hepatocellular Carcinoma (Hcc) by Adsorbing Mir-1258 and Mir-622." *Cell Cycle* 18, no. 19 (2019): 2398-413.
145. Zhao, Jing, Li Li, and Ling Peng. "Mapk1 up-Regulates the Expression of Malat1 to Promote the Proliferation of Cardiomyocytes through Pi3k/Akt Signaling Pathway." *International journal of clinical and experimental pathology* 8, no. 12 (2015): 15947.

146. Liu, Bingqi, Guangsheng Yang, Xin Wang, Jingfang Liu, Zhihua Lu, Qi Wang, Bing Xu, Zhiqian Liu, and Jie Li. "Circbach1 (Hsa_Circ_0061395) Promotes Hepatocellular Carcinoma Growth by Regulating P27 Repression Via Hur." *Journal of Cellular Physiology* 235, no. 10 (2020): 6929-41.
147. Yu, Yanhui, Lijuan Bian, Renfei Liu, Yitong Wang, and Xia Xiao. "Circular Rna Hsa_Circ_0061395 Accelerates Hepatocellular Carcinoma Progression Via Regulation of the Mir-877-5p/Pik3r3 Axis." *Cancer cell international* 21, no. 1 (2021): 1-12.
148. Zhang, Wei, Yahui Liu, Yu Fu, Wei Han, Hongji Xu, Lijia Wen, Yu Deng, and Kai Liu. "Long Non-Coding Rna Linc00160 Functions as a Decoy of Microrna-132 to Mediate Autophagy and Drug Resistance in Hepatocellular Carcinoma Via Inhibition of Pik3r3." *Cancer letters* 478 (2020): 22-33.
149. Liu, Yi, Jianchao Guo, Ka Shen, Renlong Wang, Cheng Chen, Zhiyuan Liao, and Jianbo Zhou. "Paclitaxel Suppresses Hepatocellular Carcinoma Tumorigenesis through Regulating Circ-Birc6/Mir-877-5p/Ywhaz Axis." *OncoTargets and therapy* 13 (2020): 9377.
150. Wei, GY, M Hu, L Zhao, and WS Guo. "Mir-451a Suppresses Cell Proliferation, Metastasis and Emt Via Targeting Ywhaz in Hepatocellular Carcinoma." *Eur Rev Med Pharmacol Sci* 23, no. 12 (2019): 5158-67.
